# Effects of coating composition and thickness on thermochromic performance and application in earplugs for swine fever

**DOI:** 10.1371/journal.pone.0358752

**Published:** 2026-09-22

**Authors:** Libo Wan, Junxin Luo, Jun Chen, Jing Fan, Yuebu Jike, Yuhao Luo, Danping Wang, Taoli Xiao, Yijun Liao, Yanqing Jia, Xiaoxiao Li, Kuan Peng, Xin Gao, Cheng Liu, Xiaolin Hu, Xinhai Zhou

**Affiliations:** 1 School of Digital Intelligence Design, Chengdu Technological University, Chengdu, Sichuan, China; 2 School of Materials and Environmental Engineering, Chengdu Technological University, Chengdu, Sichuan, China; 3 Sichuan Yibin Wuliangye Jingmei Printing Co., Ltd., Yibin, Sichuan, China; 4 School of Mechanical and Electrical Engineering, Yibin Industry Polytechnic College, Yibin, Sichuan, China; 5 School of Mechanical Engineering, Chengdu Technological University, Chengdu, Sichuan, China; 6 School of Robotics, Chengdu Technological University, Chengdu, Sichuan, China; 7 Chengdu Wuyu Industrial Design Co., Ltd., Chengdu, Sichuan, China; 8 School of Architecture, Southwest Minzu University, Chengdu, Sichuan, China; North-Caucasus Federal University - Pyatigorsk Campus: Severo-Kavkazskij federal’nyj universitet Patigorskij institut filial, RUSSIAN FEDERATION

## Abstract

Thermochromic coatings have been widely investigated, with most studies focusing on novel microcapsule systems. However, the thermochromic performance of coatings under a spatially separated heat source, as well as the influence of coating parameters on thermochromic behavior, remains insufficiently explored. In this study, an earplug structure embedded with a thermochromic coating was prepared, and the effects of coating filler content, coating thickness, and dye concentration on thermochromic behavior were systematically investigated. A distinct color transition was successfully achieved even when the coating was positioned away from the heat source. Simulation results indicate that, under a 40 °C heat source, the effective color‑transition temperature of the coating within the earplug structure should be maintained at approximately 34 °C. The incorporation of HBN accelerates the transition process and narrows the color-transition temperature range, enabling complete color change at around 34 °C. In addition, the effective color-changing distance is extended from 2.2 cm to 3.7 cm; however, a high HBN content reduces the ΔE color difference. Increasing coating thickness prolongs the transition time and darkens both the initial and final colors, with a thickness of 30 μm yielding the maximum ΔE value. The addition of blue dye generates visually distinguishable pre-transition and post-transition states. Although increasing blue dye concentration decreases the ΔE value between pre-transition and post-transition colors, it increases the $\Delta E^{*}$ value between the post-transition color and pig skin color. Under a 40 °C heat source, the thermochromic coating in the earplug rapidly changes from light purple to blue. These results suggest that thermochromic coatings hold potential for low-cost, visually intuitive earplugs for fever detection in swine, although further validation is required.

## 1. Introduction

In pig farming, the health of pigs is of critical importance, and an increasing number of studies have focused on these aspects [1,2]. Real-time monitoring of body temperature and early detection of febrile responses are critical for mitigating infectious disease outbreaks, safeguarding animal welfare, and improving breeding efficiency [3]. Among swine diseases, African swine fever (ASF), classical swine fever (CSF), and foot-and-mouth disease (FMD) are widely recognized as the most severe threats to pig health and production systems [4–6]. In 2019, the global economic losses attributable to ASF were estimated at approximately USD 94.5 billion [4]. Body temperature is a key physiological indicator reflecting the health status of pigs, and fever (typically ≥ 40 °C) is often an early symptom of diseases such as ASF and porcine reproductive and respiratory syndrome [7,8]. Consequently, there is an urgent need to develop efficient, accurate, and low-stress technologies for body temperature monitoring and fever warning.

Various technologies have been developed for monitoring pig body temperature and providing fever warnings, including invasive methods [7], wearable sensors [9,10], and infrared thermography (IRT) [11,12]. Among these, wearable monitoring systems such as temperature-sensing ear tags and body temperature belts offer advantages of operational simplicity and real-time data acquisition, making them suitable for large-scale farming [10]. However, existing wearable devices may cause discomfort to pigs, leading to scratching, rubbing, and other behaviors that can result in device detachment or damage. Therefore, the development of low-cost, non-invasive, and simple sensing approaches remains necessary.

In recent years, thermochromic coatings, which exhibit reversible color changes in response to temperature variation, have attracted considerable attention in the field of smart materials [13,14]. These materials have been widely applied in color-shifting actuators [15], anticounterfeiting [16,17], thermal sensing [18], smart windows [19], and solar energy storage [20,21]. Most thermochromic coatings rely on thermochromic dyes to achieve temperature-responsive chromic behavior. Thermochromic dyes are generally classified into inorganic and organic types. Inorganic thermochromic materials include VO_2_ [22], ZnO [23], and Cu_2_HgI_4_ [24], among others. However, their high phase-transition temperatures (70–800 °C), complex preparation processes, and high costs limit practical applications. In contrast, organic thermochromic dyes, typically lactone-based leuco dyes, offer advantages such as tunable color-transition temperatures, reversible bistable color switching, flexible color combinations, bright coloration, and pronounced visual response [25,26]. Most organic thermochromic dyes are currently encapsulated via microencapsulation to reduce environmental interference and improve operational stability, chromic sensitivity, and service lifetime [20]. Zhang et al. [27] fabricated thermochromic phase-change microcapsules via in-situ condensation using a ternary core composed of crystal violet lactone, bisphenol A, and phase-change wax, with a silica shell as the wall material. Liu et al. [28] developed a non-toxic chlorophenol red-water thermochromic system and its corresponding organosilicon shell microcapsules formed at the interface of W/O emulsion through reaction with octadecyltrichlorosilane, exhibiting clear and reversible color changes.

Current research on thermochromic coatings mainly focuses on building energy conservation, environmental protection, wearable textiles, and biomedical applications. However, their application in early fever warning for pigs remains insufficiently explored. Moreover, most previous studies have primarily focused on developing novel microcapsules or inorganic dyes to achieve thermochromic behavior at target temperatures, while systematic investigations of how coating composition and thickness influence thermochromic performance remain limited.

**Fig 1:**
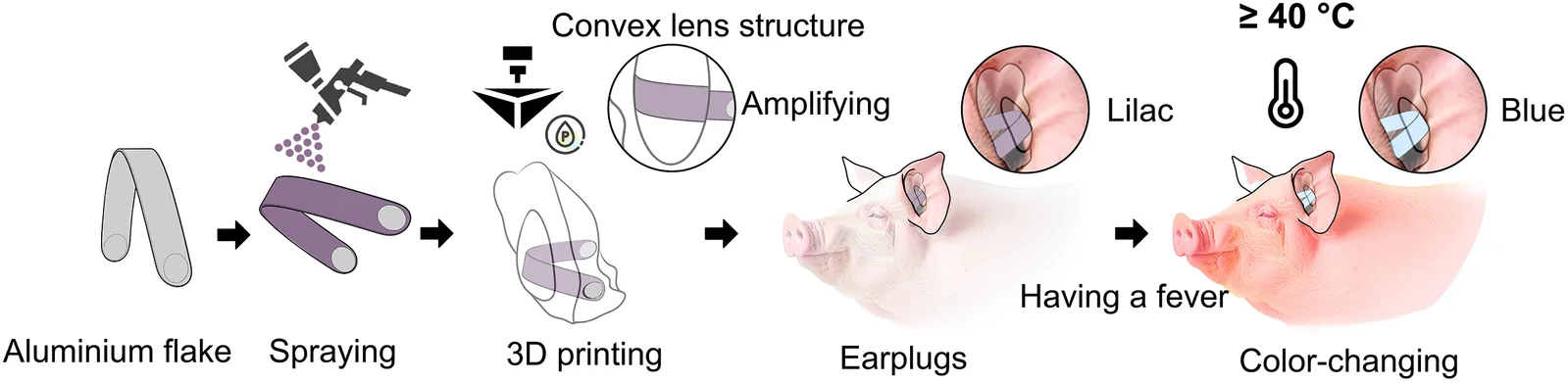
The schematic illustration of the thermochromic coating in earplug.

In this study, an earplug structure incorporating a thermochromic coating was fabricated, and the effects of coating thickness, thermally conductive fillers, and dye content on color-transition degree, color-transition temperature, and color-transition distance were systematically investigated. As shown in Fig 1, the earplug design was based on the scanned geometry of the pig ear canal to minimize discomfort, as previously validated by our research group for feasibility in pig ears [29]. The thermochromic coating was sprayed onto the central region of an aluminum sheet, which was fully encapsulated within a silicone rubber matrix fabricated via 3D printing, while one end of the aluminum strip was exposed to enable thermal contact with the heat source. A convex lens structure was integrated to magnify the coating, facilitating visual observation. Simulation results demonstrated that the combination of the thermochromic coating and earplug structure enabled a pronounced color change at 40 °C and was successfully demonstrated in pig ear applications. This work is expected to provide a non-invasive, real-time, and low-cost strategy for pig body temperature monitoring.

## 2. Experimental

### 2.1. Materials

An aqueous polyurethane emulsion (F0401, purity: 38 wt.%) was supplied by Shenzhen Jitian Chemical Co., Ltd., Shenzhen, China. Thermochromic microcapsules (TM, Chameleon TP-Red 33), exhibiting a color transition from red to colorless at 33 °C, were supplied by Color Change Technology Co., Ltd., Shenzhen, China. The shell material of TM consists of polyoxymethylene melamine, and the emulsifier is a styrene-maleic anhydride monomethyl maleate copolymer. The core material of TM is composed of methyl stearate, ethyl stearate, Orange DCF, and Red DCF. Hexagonal boron nitride (HBN, d:25 nm, 99.9%, SSA:4.6 m^2^/g) was supplied by Suzhou Yananometer New Materials Technology Co., Ltd., Suzhou, China. Blue dye (BD, Blue SG2050, phthalocyanine blue, purity: 48 wt.%) was supplied by Beijing Maier Chemical Technology Co., Ltd., Beijing, China. 3-aminopropyltrimethoxysilane (purity>98.5%) was purchased from Aladdin Industrial Corporation, Shanghai, China. Pig head samples were obtained from a commercial food market in Chengdu, China. The animals were originally raised and slaughtered for food consumption rather than for research purposes. Since all tissues were derived from postmortem specimens intended for human consumption, no antemortem intervention or euthanasia was involved in this study. Accordingly, this work was exempt from approval by the Institutional Animal Care and Use Committee. After procurement, all samples were handled in accordance with institutional biosafety guidelines for postmortem biological materials.

### 2.2. Preparation of PU/TM/HBN/BD coating

HBNs were initially dispersed in a water-ethanol solution (5/95 vol%) containing 3-aminopropyltrimethoxysilane, followed by ultrasonication for 10 min and mechanical stirring at 70 °C. After 2 h of stirring, the resulting suspension was filtered and subsequently dried at 80 °C for 24 h.

Thermochromic powder was first incorporated into the polyurethane emulsion and mechanically stirred for 10 min. Subsequently, the modified HBNs and blue dye were added, followed by mechanical stirring for 20 min and ultrasonication for 5 min to ensure uniform dispersion. The final mixture was then sprayed onto aluminum plates. Control samples, including pure polyurethane emulsion and samples containing only thermochromic microcapsules, were also prepared for comparison. The detailed compositions are summarized in Table 1. The samples were labeled as PU and PU/TM/HBN-x/BD-y, where x represents the HBN content and y represents the blue dye content. The earplugs were fabricated by Dongguan Yijia Handboard Model Co., Ltd. (Dongguan, China) using a combination of spraying and 3D printing techniques.

**Table 1:** The specific composition of PU/TM/HBN/BD coatings.

Specimens	TM (wt.%)	HBN (wt.%)	BD (wt.%)	Polyurethane emulsion (wt.%)
PU	0	0	0	100
PU/TM	12	0	0	88
PU/TM/HBN-3	12	3	0	85
PU/TM/HBN-6	12	6	0	82
PU/TM/HBN-12	12	12	0	76
PU/TM/BD-2.4	12	0	2.4	85.6
PU/TM/HBN-12/BD-0.12	12	12	0.12	75.88
PU/TM/HBN-12/BD-0.24	12	12	0.24	75.76
PU/TM/HBN-12/BD-0.6	12	12	0.6	75.4

### 2.3. Material characterization

The morphology of the PU/TM/HBN-x/BD-y composites was characterized using scanning electron microscopy (SEM, FEI inspect F50, Thermo Scientific, USA). The functional groups were analyzed using Fourier transform infrared spectroscope (FTIR, Nicolet, 170SX, Wisconsin, USA) over a wavenumber range of 400–4000 cm^−1^. The crystalline phases of the PU/TM/HBN-x/BD-y composites were examined using an X-ray diffractometer (XRD, Philips PC-APD). The scanning 2θ range was 5–60°, with a scan rate of 4°/min. The thermal conductivity of the composites was measured using a laser flash thermal conductivity analyzer (NETZSCH, LFA467, Germany).

### 2.4. Thermochromic performance test

The temperature of the plate-type heater was set to 33, 34, 35, 36 and 37 °C, respectively. When the target temperature was reached, the sample was placed onto the heater and timing was initiated. The measurement was terminated when the coating completed its color transition and reached a stable state. The entire process was recorded by photography, and the surface temperature was monitored using an infrared thermal imaging instrument. A CIE standard D65 illuminant (6500 K, simulating average daylight) was used as the sole light source. The camera was fixed on a tripod, positioned perpendicular to the sample surface at a constant distance of 50 cm. The camera settings were kept unchanged throughout all measurements: ISO 100, aperture f/8, shutter speed 1/60 s, and manual white balance. All samples were recorded under identical ambient conditions at the same time to minimize errors caused by variations in environmental lighting. Each experiment was repeated three times. The thermochromic stability of the coating was evaluated through 100 cycles of heating-cooling experements between 25 °C and 34 °C.

The changes in L, a, and b chromaticity values during the color transition of the coating were analyzed using Adobe Photoshop CS software. A fixed circular region of interest of 100 × 100 pixels was selected at the center of each sample for color analysis. The total color difference (ΔE) was calculated based on the CIE Lab color space, according to the following equation:


$$\begin{array}{c} \Delta E=\sqrt{(\Delta L^{\text{2}}+\Delta a^{\text{2}}+\Delta b^{\text{2}})} \end{array}$$

where ΔL represents the change in lightness, Δa represents the change between red and green, and Δb represents the change between yellow and blue.

To evaluate the achievable color-transition distance of the thermometric coating under a unidirectional heat source, one end of the aluminum sheet coated with the thermochromic layer was placed on a 40 °C heater, and a ruler was positioned above the sample. The entire process was recorded photographically, and the surface temperature was monitored using an infrared thermal imager. Each experiment was repeated three times.

### 2.5. Simulation experiment on pig ears

The simulation experiments were conducted using a self-developed device on a postmortem pig head obtained from a commercial food market. First, a heating plate was attached to the inner surface of the pig ear canal wall. Subsequently, the earplug was inserted into the pig ear. The heating plate was connected to a power supply, and the temperature was set at 38, 39, 40, 41, and 42 °C, respectively. The color changes of the coating were recorded by photography throughout the experiment. Each test was repeated three times.

### 2.6. Simulation of heat conduction behavior of earplugs

To quantitatively analyze the steady-state temperature field distribution in the intermediate color-transition region of the earplug, a three-dimensional transient heat transfer simulation was performed using COMSOL Multiphysics® software. The model was developed based on the Heat Transfer in Solids module, and the boundary conditions were defined as follows: both surfaces of the aluminum sensing base (i.e., the aluminum strip in the earplug in contact with the ear canal wall) were maintained at 38 and 40 °C, while the outer surfaces were assumed to be exposed to a natural heat dissipation environment. The material properties were assigned based on experimental measurements. The thermal conductivity, specific heat capacity, and density of silicone rubber were 0.5 W/(m·K), 1900 J/(kg·K), and 1100 kg/m^3^, respectively. For the aluminum sheet, the corresponding values were 238 W/(m·K), 900 J/(kg·K), and 2700 kg/m^3^, respectively. Considering the weak airflow within the ear canal and the confined space after earplug assembly, convective heat transfer was neglected, and only surface thermal radiation was considered. The emissivity of the rubber outer surface was set to 0.95 [30], and the ambient temperature was maintained at 20 °C.

## 3. Results and discussion

### 3.1. Simulation results for the earplug

**Fig 2:**
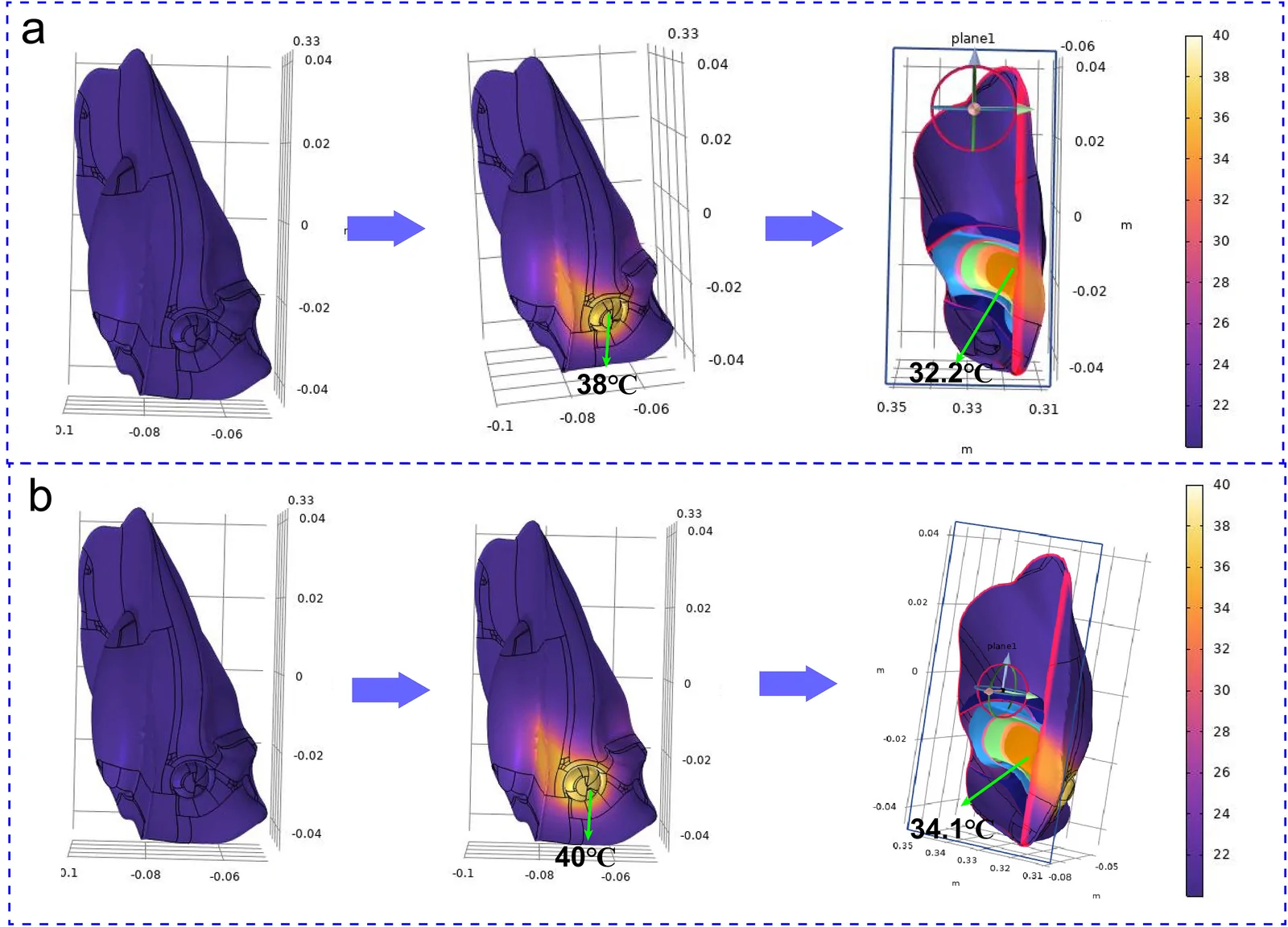
The simulation results of earplugs with a heat source temperature of: (a) 38 °C; (b) 40 °C.

Fig 2 shows the temperature distribution of the aluminum sheet embedded in silicone rubber under different heat sources simulated using COMSOL Multiphysics®. The heat source temperatures applied to the exposed portion of the aluminum strip were set at 38 °C and 40 °C, corresponding to normal and febrile body temperatures in most pigs. As shown in the earplug structure (Fig 1), a certain distance exists between the heat source and the visible region of the earplug, indicating that the thermochromic coating is not in direct contact with the heat source. As illustrated in Fig 2a, when the heat source temperature was 38 °C, the central region of the aluminum sheet reached 32.2 °C; when the heat source temperature was increased to 40 °C, the corresponding temperature increased to 34.1 °C. Based on these simulation results, the color-transition temperature of the thermochromic coating should be controlled at approximately 34 °C, slightly lower than the simulated reference value of 34.1 °C. However, commercially available thermochromic microcapsules typically exhibit color-transition temperatures of 31, 33, and 35 °C, none of which precisely matches the target value of 34 °C. In previous studies, including our own, a delayed response in the color-transition behavior of microcapsules has frequently been observed. Therefore, microcapsules with a phase-transition temperature of 33 °C were selected as the primary filler for the thermochromic coating.

### 3.2. Characterization of PU/TM/HBN/BD coatings

**Fig 3:**
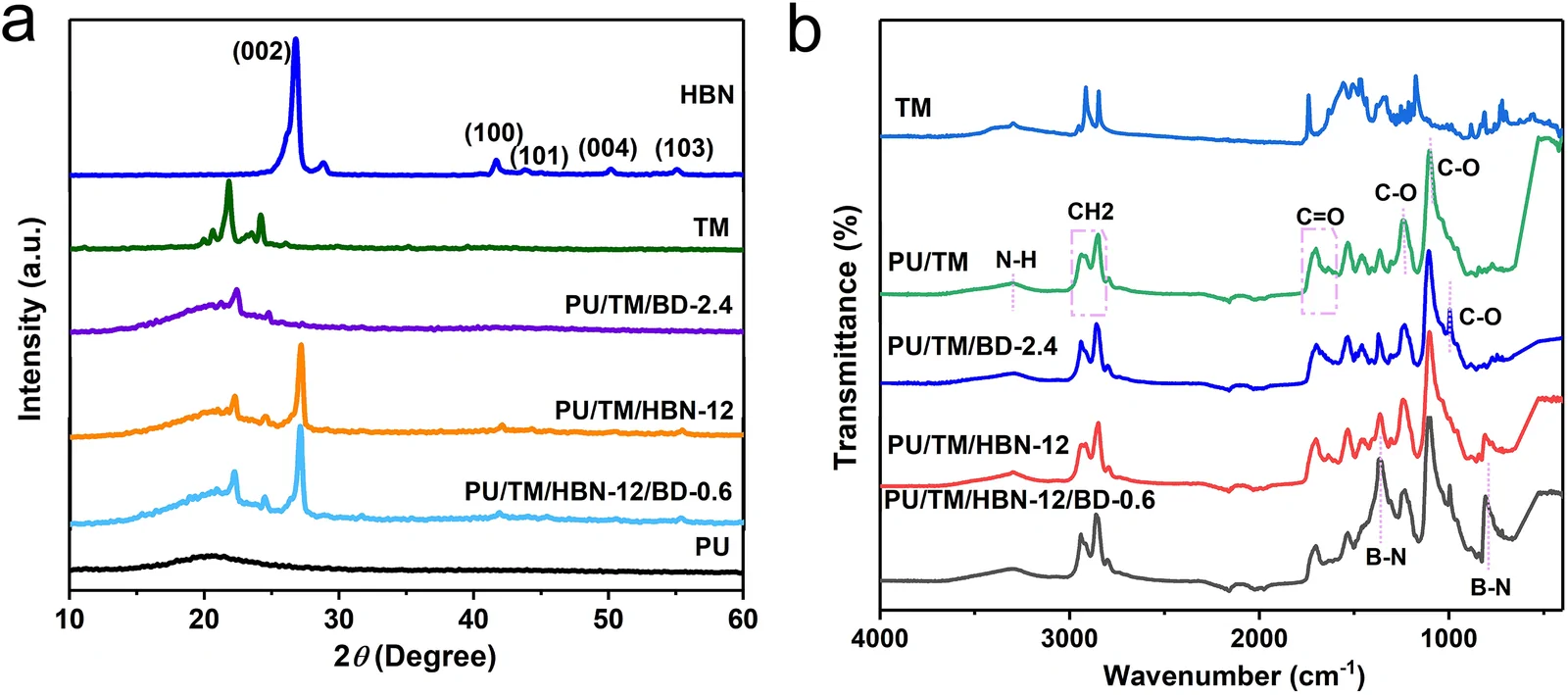
(a) The XRD patterns of the PU/TM/HBN/BD coatings; (b) The FTIR spectra of the PU/TM/HBN/BD coatings.

Fig 3a shows the XRD patterns of the PU/TM/HBN-x/BD-y coatings (where x represents the HBN content and y represents the blue dye content). The diffraction patterns of HBN, TM, and neat PU resin were also analyzed for comparison. A broad diffraction peak in the range of 2θ about 17−23° is observed for neat PU, indicating its amorphous nature [31,32]. For pure TM, three characteristic diffraction peaks appear at 2θ about 20.6°, 21.8°, and 24.1°, which are also present in the PU/TM/BD-2.4, PU/TM/HBN-12, and PU/TM/HBN-12/BD-0.6 coatings, confirming the successful incorporation of TM. For HBN, five characteristic peaks are observed at 2θ about 26.7°, 41.7°, 43.9°, 50.1°, and 55.2°, corresponding to the (0 0 2), (1 0 0), (1 0 1), (0 0 4), and (1 0 3) crystal planes, respectively [33]. These diffraction peaks are also detected in the PU/TM/HBN-12 and PU/TM/HBN-12/BD-0.6 coatings, further confirming the presence of HBN. No distinct diffraction peaks of the blue dye are observed in PU/TM/BD-2.4 and PU/TM/HBN-12/BD-0.6, which may be due to the small amount of the blue dye or its amorphous phase.

Fig 3b shows the FTIR spectra of the PU/TM/HBN-x/BD-y coatings. As shown in Fig 3b, all samples exhibit a broad absorption band at 3300–3400 cm^-1^, corresponding to N-H stretching vibrations of PU [34] and polyoxymethylene melamine (shell material of TM). The bands at 2920 and 2850 cm^-1^ are assigned to the asymmetric and symmetric stretching vibrations of -CH_2_ groups in PU [34] and methyl stearate (core material of TM) [35]. The absorption peak at 1740 cm^-1^ corresponds to C = O stretching vibrations of urethane groups in PU and ester groups in methyl stearate [35]. The bands at 1230−1250 and 1080−1100 cm^-1^ are attributed to C-O stretching vibrations of PU. For the PU/TM/HBN-12 and PU/TM/BD/HBN-12/BD-0.6 coatings, the peaks at 1370 cm^-1^ and 780 cm^-1^ are assigned to in-plane stretching and out-of-plane bending vibrations of B-N bonds, respectively, confirming the successful incorporation of HBNs [36]. In PU/TM/HBN-12/BD-0.6 and PU/TM/BD-2.4, an additional absorption peak appears at approximately 1000 cm^-1^, which is attributed to C-O-related vibrations of the blue dye.

**Fig 4:**
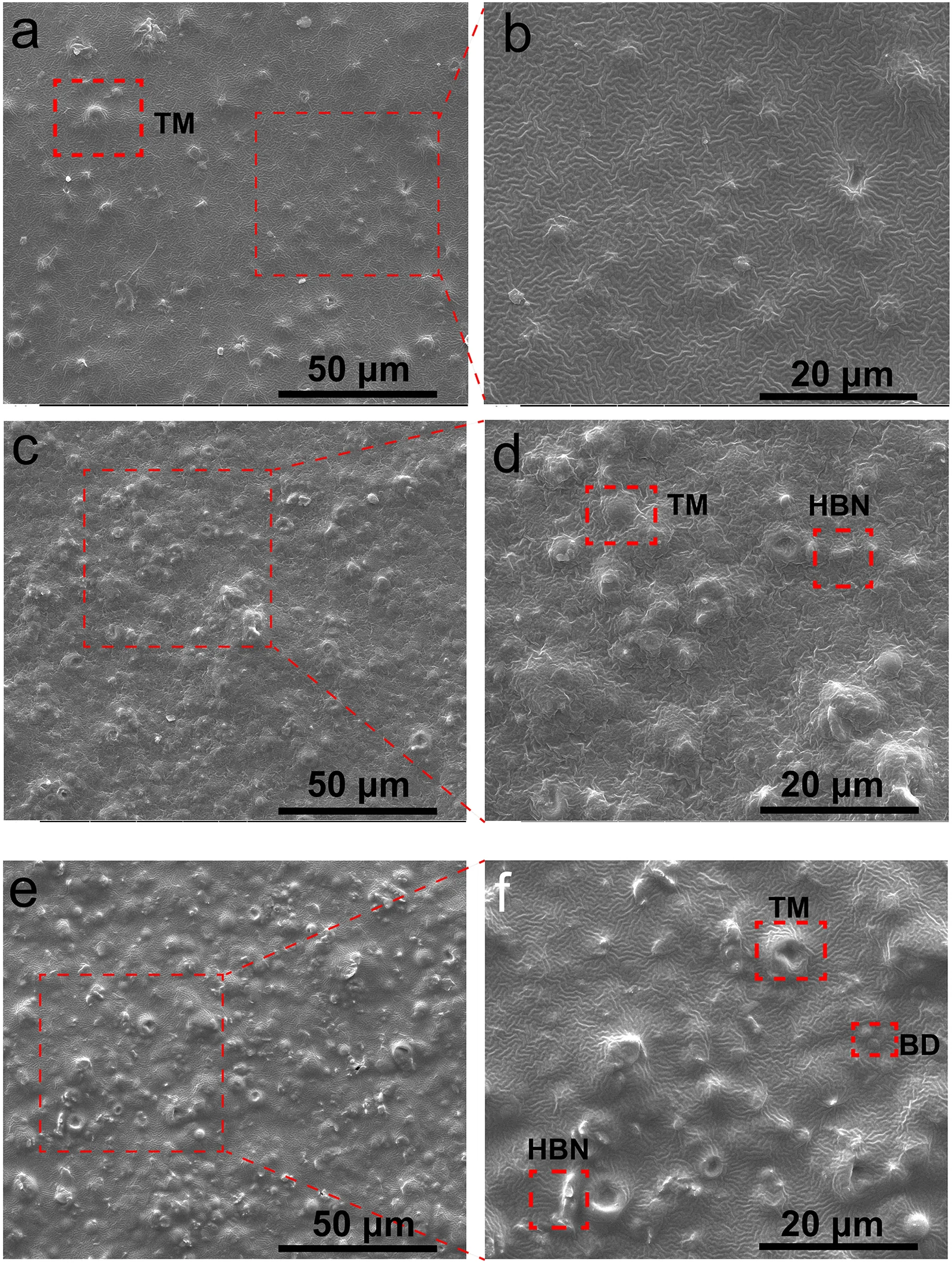
The surface morphology of: (a, b) PU/TM; (c, d) PU/TM/HBN-12; (e, f) PU/TM/HBN-12/BD-0.6 coatings.

Fig 4 shows the surface morphology of the PU/TM, PU/TM/HBN-12, and PU/TM/HBN-12/BD-0.6 coatings. Fig 4a, b present the morphology of the PU/TM coating. It can be observed that the thermochromic microcapsules are well compatible with the PU resin matrix, with no obvious cracks or interfacial voids, and are uniformly dispersed throughout the matrix. Fig 4c, d show the surface morphology of the PU/TM/HBN-12 coating. Compared with PU/TM, numerous small protrusions are observed, which are likely attributed to HBN flakes. These flakes exhibit good compatibility with the PU matrix and are tightly embedded within the resin, resulting in an indistinct flake structure. In the PU/TM/HBN-12/BD-0.6 coating (Fig 4e, f), some smaller microspheres, smaller than the thermochromic microcapsules, are observed, which are likely associated with the blue dye particles. In all coatings, neither HBN nor the dye-derived microspheres show obvious agglomeration, and both are uniformly distributed within the PU matrix.

### 3.3. Thermochromic property of PU/TM/HBN/BD coatings

**Fig 5:**
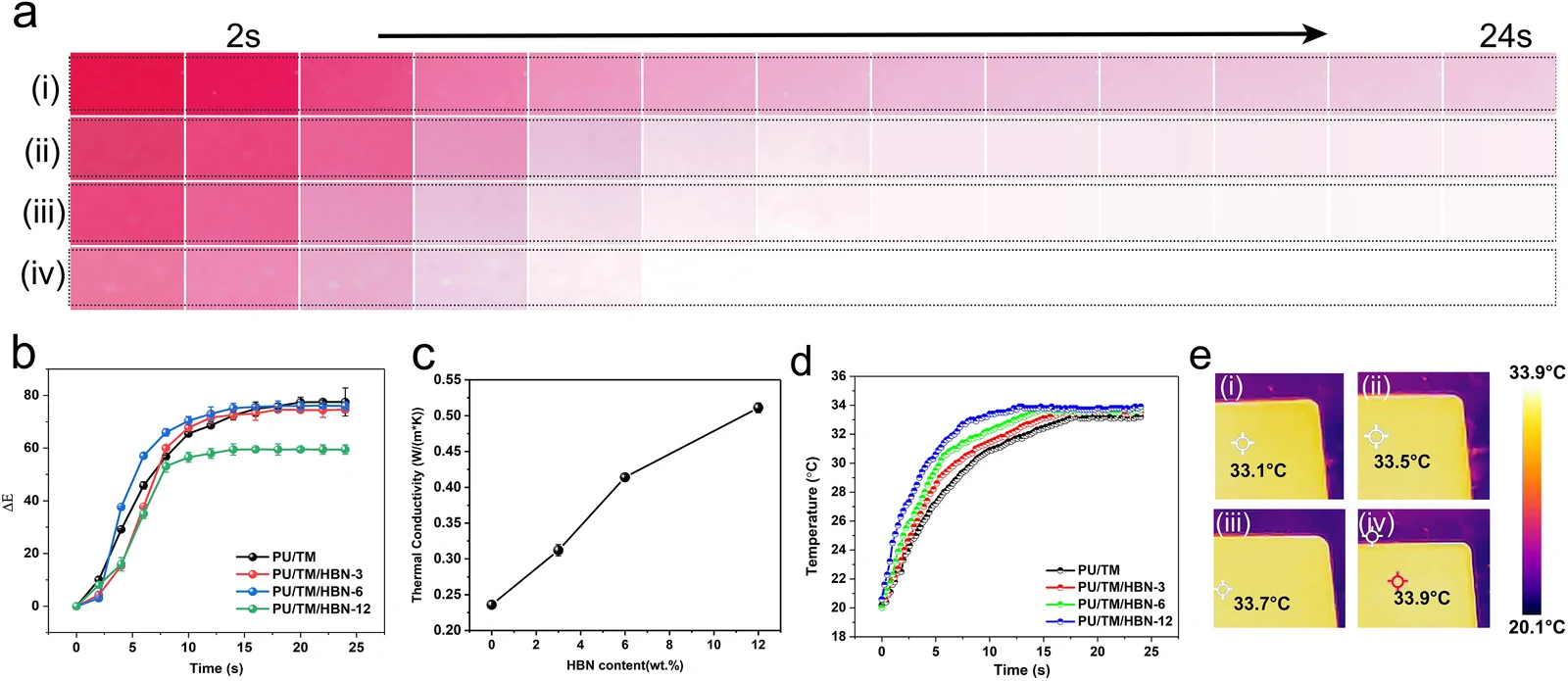
(a) The thermochromic photos with the time for (i) PU/TM, (ii) PU/TM/HBN-3, (iii) PU/TM/HBN-6, (iv) PU/TM/HBN-12 coatings; (b) The color difference (Δ𝐄) calculated based on the initial chromaticity values; (c) The thermal conductivity; (d) The surface temperature variation with time; (e) Infrared images of PU/TM, PU/TM/HBN-3, PU/TM/HBN-6, and PU/TM/HBN-12 coatings.

To systematically investigate the effect of HBNs on the thermochromic performance of the coating, different contents of HBNs were incorporated into the thermochromic coating. Thermochromic behavior was evaluated at 34 °C, and the relationship between color transition and coating surface temperature was analyzed. Fig 5a presents thermochromic images of PU/TM, PU/TM/HBN-3, PU/TM/HBN-6, and PU/TM/HBN-12 coatings over time. It can be observed that the PU/TM coating begins to change color at 2 s, and the color-transition stabilization time is about 20 s. The color changes from deep red to light pink, but does not fully become colorless. With increasing HBNs content, the color-transition stabilization time decreases. Specifically, the stabilization times are approximately 18, 16, and 14 s for PU/TM/HBN-3, PU/TM/HBN-6, and PU/TM/HBN-12 coatings, respectively, which is consistent with the color difference (ΔE) results shown in Fig 5b. This behavior is mainly attributed to the enhanced thermal conductivity of the coating induced by HBNs addition [37,38], as shown in Fig 5c. It is also evident that, with increasing HBNs content (Fig 5d), the surface temperature rise rate of the coating increases accordingly. The surface temperatures of PU/TM, PU/TM/HBN-3, PU/TM/HBN-6, and PU/TM/HBN-12 stabilize at approximately 18, 17, 15, and 13 s, respectively.

Furthermore, both the initial and final stable colors of the coating become lighter with increasing HBNs content. The lighter initial color is likely due to the intrinsic white color of HBNs. The lighter final stable color may be attributed not only to the inherent whiteness of HBNs but also to the surface temperature of the coating. As shown in Fig 5d, e, the stabilized temperatures are approximately 33.2 ± 0.79, 33.5 ± 0.53, 33.7 ± 0.52, and 33.9 ± 0.61 °C for PU/TM, PU/TM/HBN-3, PU/TM/HBN-6, and PU/TM/HBN-12 coatings, respectively. In Fig 5a, the stable color of PU/TM/HBN-12 is white (L = 100, a = 0, b = 0), indicating a complete color transition. The actual temperature at which complete discoloration occurs corresponds to a surface temperature of approximately 33.9 ± 0.61 °C. However, the ΔE value of PU/TM/HBN-12 is the lowest among the four samples, which is associated with the fact that HBNs addition lightens the initial color. Notably, unlike the other coatings, PU/TM/HBN-12 enables a complete transition from red to colorless.

**Fig 6:**
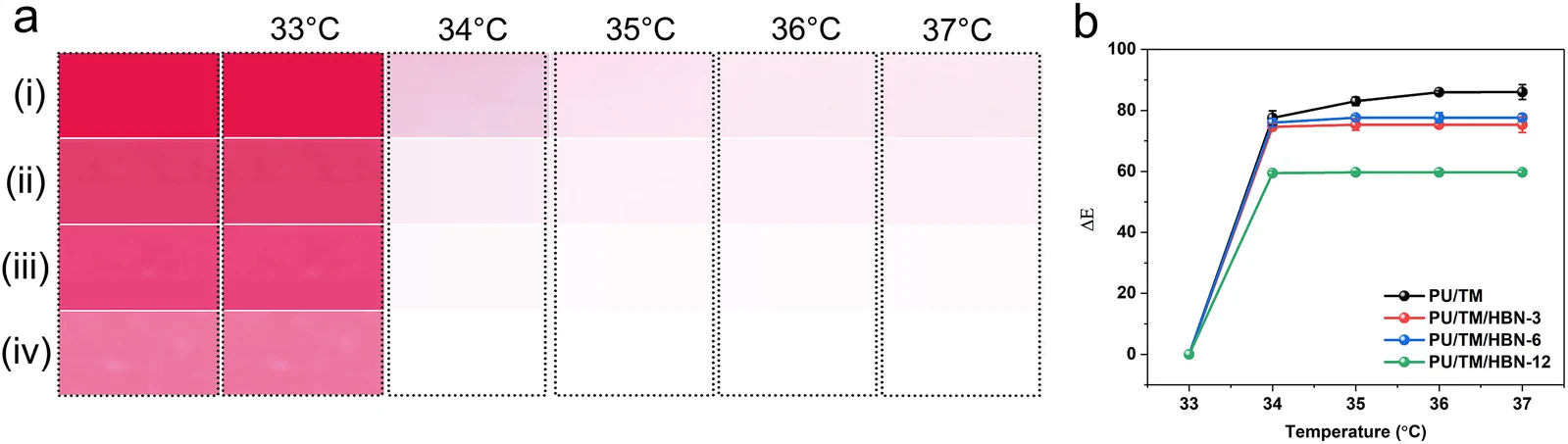
(a) The thermochromic photos at different temperature of heat source for (i) PU/TM, (ii) PU/TM/HBN-3, (iii) PU/TM/HBN-6, (iv) PU/TM/HBN-12 coatings; (b) The color difference calculated based on the initial chromaticity values of PU/TM, PU/TM/HBN-3, PU/TM/HBN-6, and PU/TM/HBN-12 coatings.

Fig 6 shows the thermochromic images and corresponding color difference values of PU/TM, PU/TM/HBN-3, PU/TM/HBN-6, and PU/TM/HBN-12 coatings under a heat source temperature range of 33−37 °C. It can be observed that none of the four coatings exhibit color transition at 33 °C, whereas the onset of color change occurs at 34 °C. For the PU/TM coating, the color gradually lightens with increasing temperature and stabilizes at 36 °C. For the PU/TM/HBN-3 and PU/TM/HBN-6 coatings, the stable color at 35 °C is lighter than that at 34 °C, while no significant color variation is observed between 35, 36, and 37 °C, indicating that the color transition becomes stable at approximately 35 °C. For the PU/TM/HBN-12 coating, complete color transition occurs at around 34 °C. These results indicate that increasing HBNs content narrows the temperature range of the color transition and improves the temperature response sensitivity of the coating.

**Fig 7:**
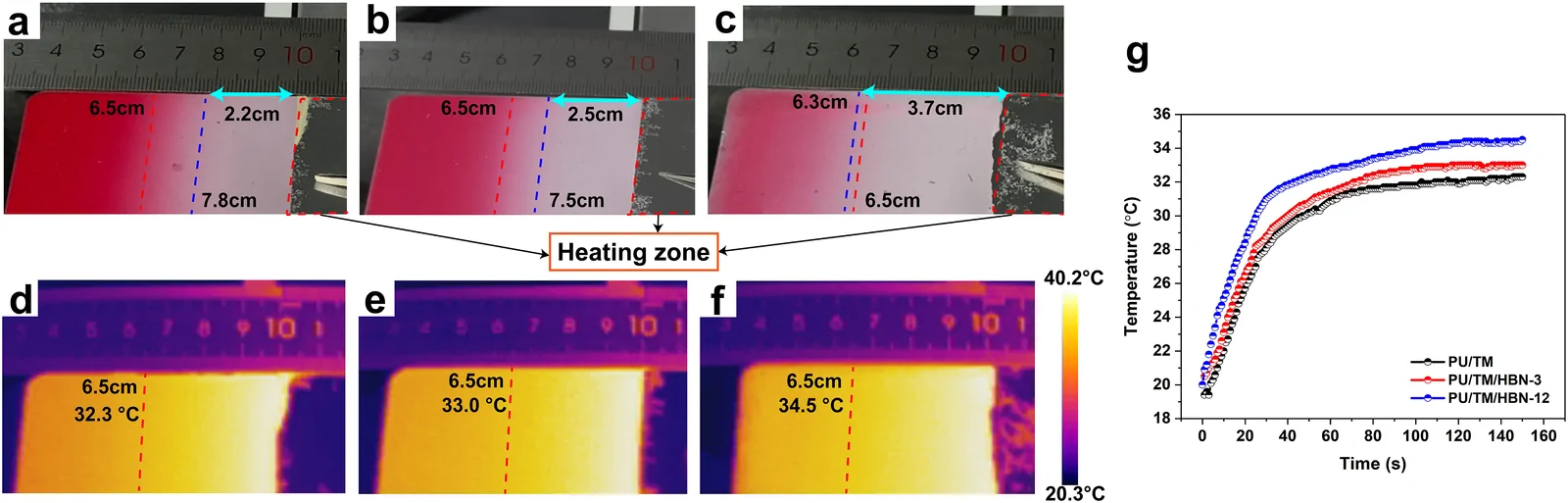
The thermochromic photos of: (a) PU/TM; (b) PU/TM/HBN-3; (c) PU/TM/HBN-12 coatings; The Infrared images of: (d) PU/TM; (e) PU/TM/HBN-3; (f) PU/TM/HBN-12 coatings; (g) The surface temperature variation with time for PU/TM, PU/TM/HBN-3, and PU/TM/HBN-12 coatings.

To further verify the above results and evaluate the color-transition distance of the thermochromic coating under a 40 °C heat source, one end of an aluminum sheet coated with the thermochromic layer was placed on a 40 °C heater, as shown in Fig 7a-c. It can be observed that the complete color-transition distance increases with increasing HBNs content. Specifically, the distances are approximately 2.2 ± 0.10 cm, 2.5 ± 0.10 cm, and 3.7 ± 0.10 cm for PU/TM, PU/TM/HBN-3, and PU/TM/HBN-12 coatings, respectively. This behavior is primarily attributed to the enhanced thermal conductivity introduced by HBNs [37,39], which leads to higher coating temperatures at the same spatial position, as shown in Fig 7d-f. At the 6.5 cm position (Fig 7g), the temperatures of the PU/TM, PU/TM/HBN-3, and PU/TM/HBN-12 coatings stabilize at approximately 32.3 ± 0.36 °C, 33 ± 1.13 °C, and 34.5 ± 1.08 °C, respectively. These results indicate that the incorporation of HBNs effectively increases the color-transition distance to 3.7 cm, thereby enabling color change at a defined distance from the heat source and meeting the requirements of practical application scenarios.

**Fig 8:**
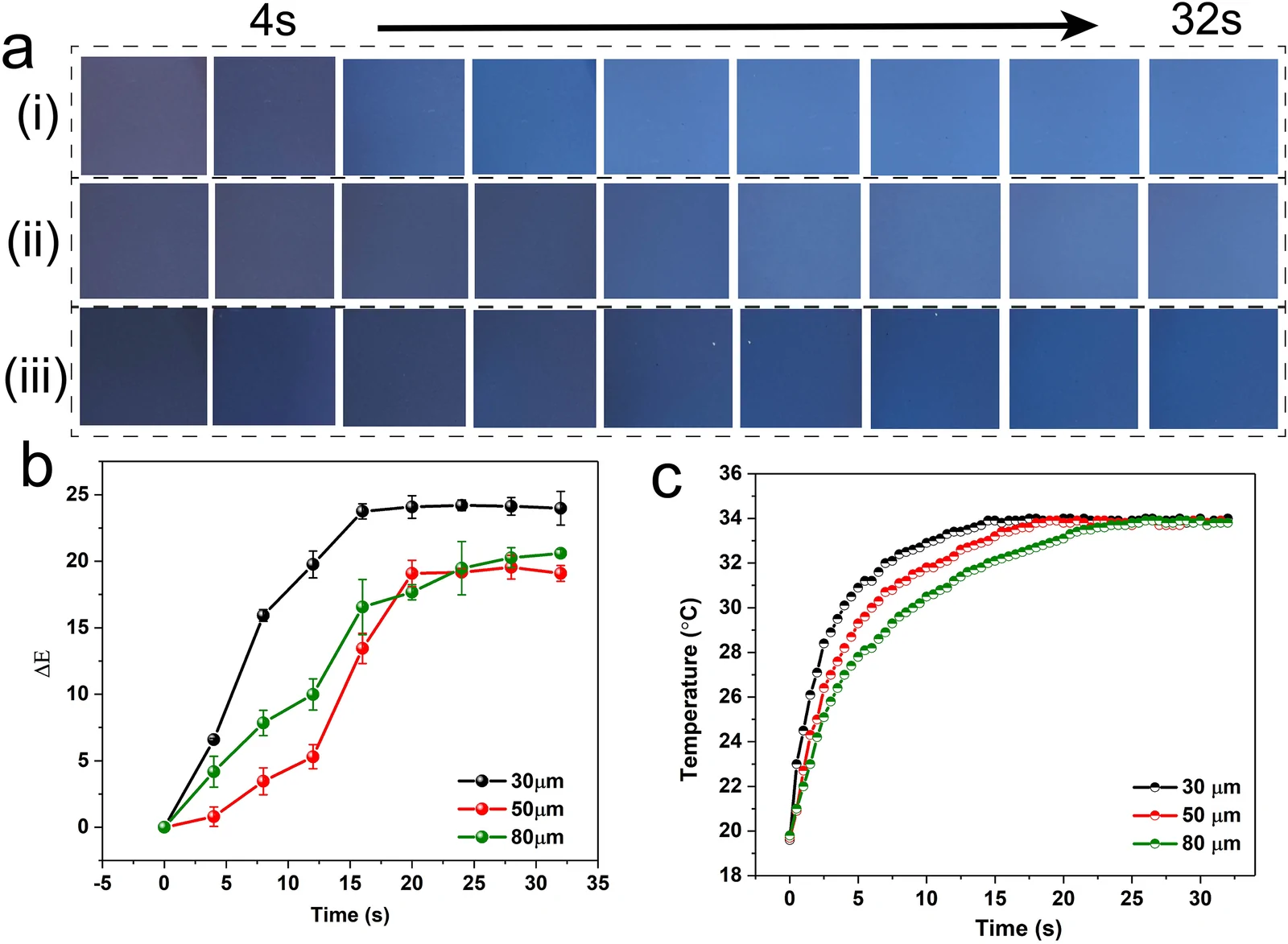
(a) The thermochromic photos as a function of time for the PU/TM/BD-2.4 samples with the thickness of: (i) 30 μm, (ii) 50 μm, (iii) 80 μm; (b) The color difference calculated based on the initial chromaticity values; (c) The surface temperature variation with time for the PU/TM/BD-2.4 samples with the thickness of 30 μm, 50 μm, and 80 μm.

To investigate the effect of coating thickness on color transition, thermochromic coatings with thicknesses of 30, 50, and 80 μm were prepared, and their thermochromic behavior and surface temperature were evaluated under a 34 °C heat source. Blue dye was incorporated into this group of coatings, resulting a blue appearance, as shown in Fig 8a. With increasing thickness, the time required for complete color transition increases. The complete color-transition times are 16 s, 20 s, and 28 s for the 30, 50, and 80 μm coatings, respectively. This trend is also reflected in the time-dependent ΔE results (Fig 8b), which is primarily attributed to the slower surface temperature rise with increasing thickness, as shown in Fig 8c. In addition, both the initial and final stable colors become darker with increasing thickness, which can be attributed to enhanced light absorption and scattering. Notably, among the three samples, the 30 μm coating exhibits the highest ΔE value (Fig 8b). However, ΔE is not linearly proportional to thickness. The ΔE value of the 50 μm sample is significantly lower than that of the 80 μm sample during the first 20 s, while the difference becomes negligible in the last 12 s. This behavior may be associated with optical absorption, scattering, and thermal conduction effects. According to the Kubelka-Munk theory [40], we propose the following explanation: the ΔE of a thermochromic coating depends not only on the number of activated dye molecules but also on light absorption and scattering processes. Previous studies have also reported a non-linear relationship between coating thickness and optical properties [41,42]. During the first 20 s, the 80 μm coating has a thicker uncoloured surface layer than the 50 μm coating due to its lower surface temperature (Fig 8c). Incident light is strongly scattered by this uncoloured layer; part of the scattered light is directly reflected back to the observer. The remaining transmitted light reaches the coloured bottom layer and is partially absorbed. The unabsorbed portion then re-enters the uncolored top layer and undergoes additional scattering before exiting the coating. This multiple-scattering process enhances the overall reflectance measured from the surface, leading to a higher ΔE value for the 80 μm coating despite its lower surface temperature compared with the 50 μm sample [43]. After approximately 20 s, the surface temperature difference between the two coatings becomes small. At this stage, the previously uncolored surface layer of the 80 μm coating also warms and undergoes color transition, reducing its scattering contribution. Consequently, the ΔE values of the two coatings converge and show no significant difference. This phenomenon requires further investigation in future work. Overall, a thickness of 30 μm enables faster color transition and a relatively higher ΔE value, making it the more suitable thickness for thermochromic coatings.

**Fig 9:**
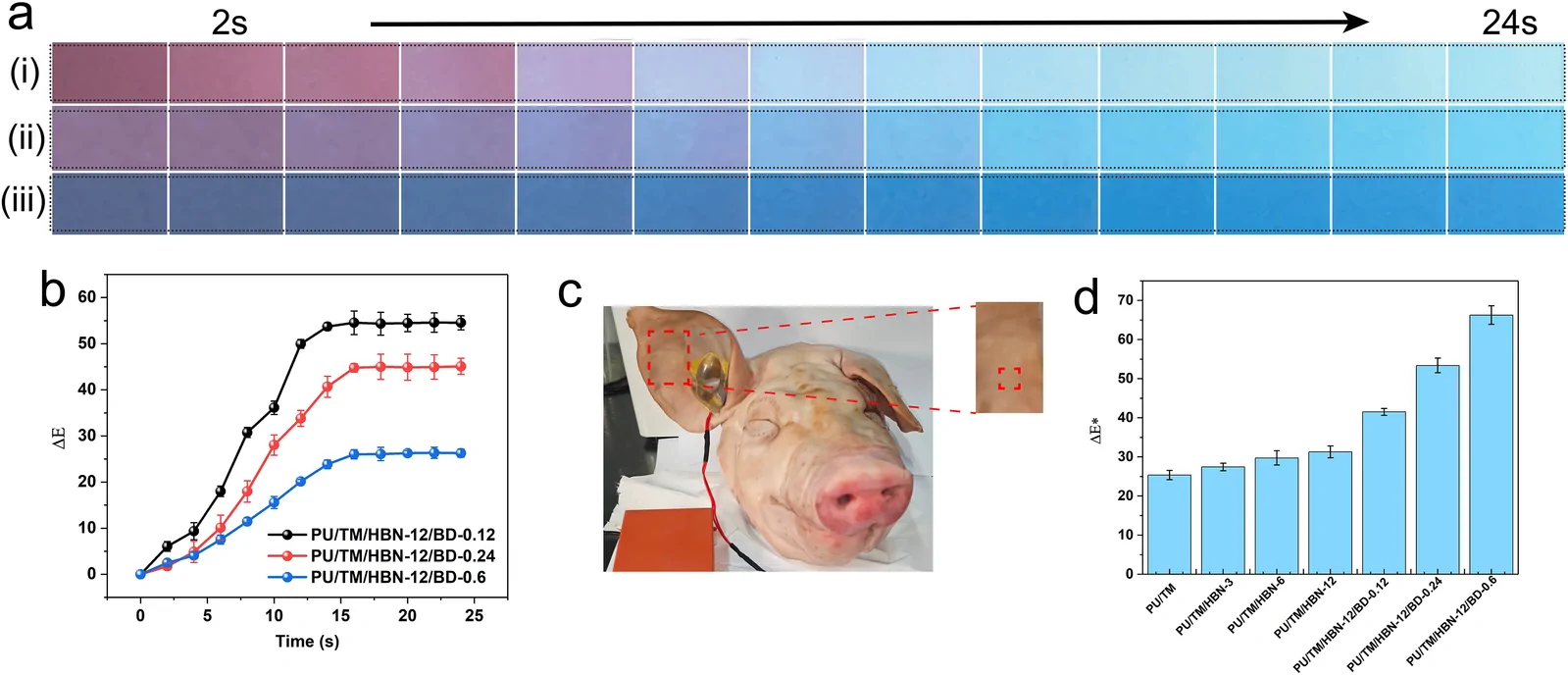
(a) The thermochromic photos as a function of time for: (i) PU/TM/HBN-12/BD-0.12, (ii).

Due to the limited distinguishability of a single color transition, blue dye was incorporated into the coatings. To investigate the effect of blue dye concentration on the ΔE value, three coatings with different dye contents were prepared, namely PU/TM/HBN-12/BD-0.12, PU/TM/HBN-12/BD-0.24, and PU/TM/HBN-12/BD-0.6, as shown in Fig 9. The initial colors of the coatings are dark red, purple, and deep blue at blue dye concentrations of 0.12, 0.24, and 0.6 wt.%, respectively (Fig 9a). The corresponding final stable colors are light blue, bright blue, and pure blue, respectively. The stabilization time of all three groups is approximately 14 s, indicating that the addition of blue dye has a negligible effect on thermal conductivity and color-transition kinetics. In addition, as shown in Fig 9b, the ΔE values decrease with increasing blue dye concentration, suggesting that excessive dye loading is not favorable for optimizing color contrast. Furthermore, to evaluate the potential application of these coatings for pig ear monitoring, the ΔE values between the post-transition coating color and the reference color of pig ears were also calculated (Fig 9d). It is noteworthy that the ΔE values of all blue-dye-containing coatings are significantly higher than those of coatings without blue dye. Moreover, the ΔE values increase with increasing blue dye concentration, indicating the necessity of incorporating blue dye for practical visual recognition. Overall, when the blue dye concentration is 0.24 wt.%, the color contrast before and after transition is pronounced, while the color difference between the post-transition state and pig ear color is relatively large. It’s worth mentioning that this formal inferential testing was not performed and that quantitative comparisons are therefore described descriptively rather than as statistically significant differences.

PU/TM/HBN-12/BD-0.24, (iii) PU/TM/HBN-12/BD-0.6; (b) The color difference calculated based on the initial chromaticity values; (c) Photos of a pig’s head and its skin color; (d) The color difference calculated based on the skin color of pig’s head for PU/TM/HBN/BD coatings.

**Fig 10:**
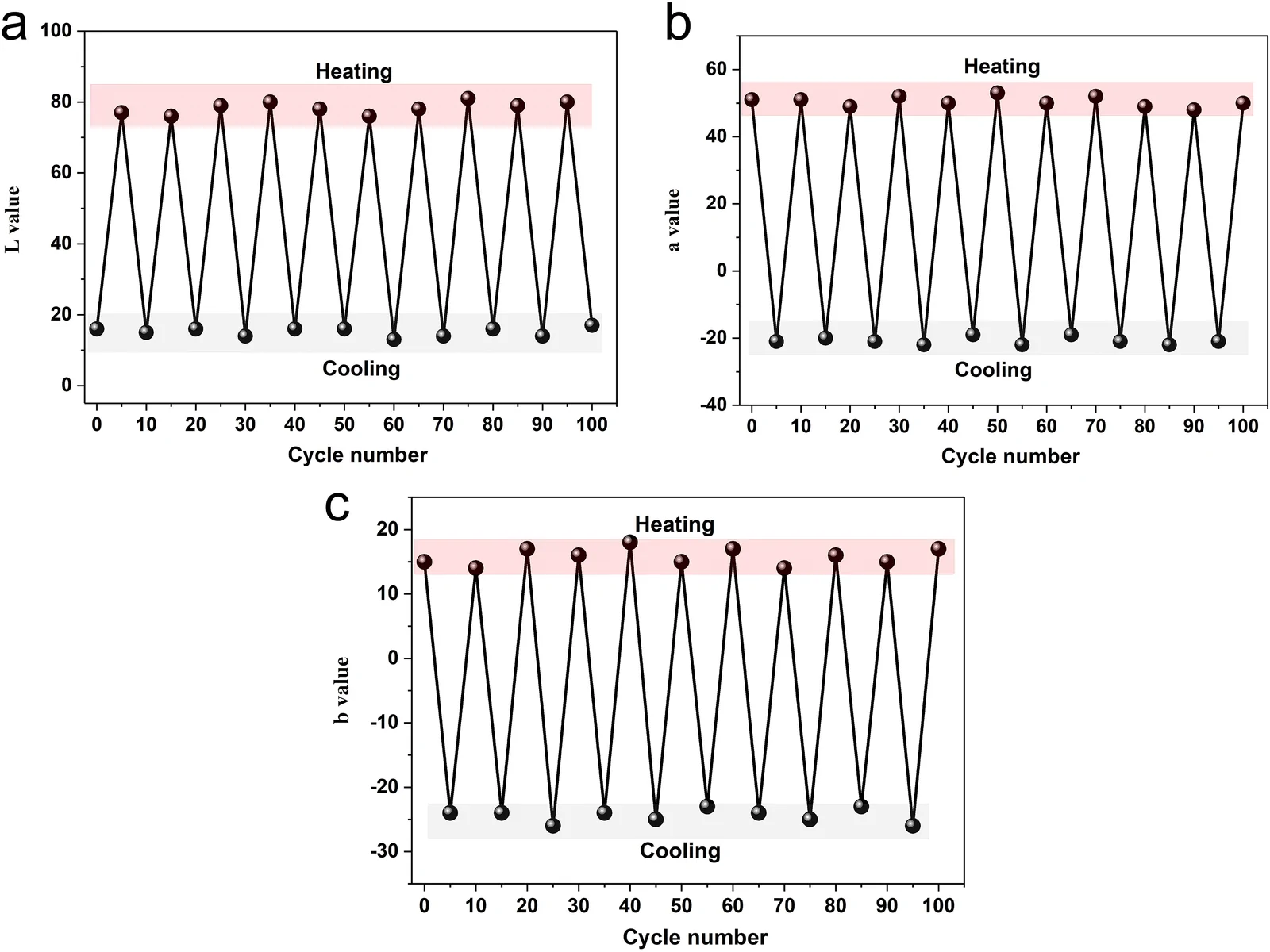
The measured values of the PU/TM/hBN‑12/BD‑0.24 thermochromic coating under 100 heating‑cooling cycles: (a) L value, (b) a value, and (c) b value.

To evaluate the thermochromic stability and practical applicability of the coating, repeated heating-cooling cycle tests were conducted between 25 °C and 34 °C. Since the PU/TM/hBN‑12/BD‑0.24 coating exhibited the best overall performance, it was selected for this evaluation. As shown in Fig 10, after 100 cycles, the L, a, b values show only minor fluctuations without any significant variation. This indicates that the coating possesses good colour reversibility and structural stability. Furthermore, it suggests that the incorporation of HBNs and blue dye does not noticeably affect the thermochromic reversibility or long-term stability.

### 3.4. Simulation result on pig ears

**Fig 11:**
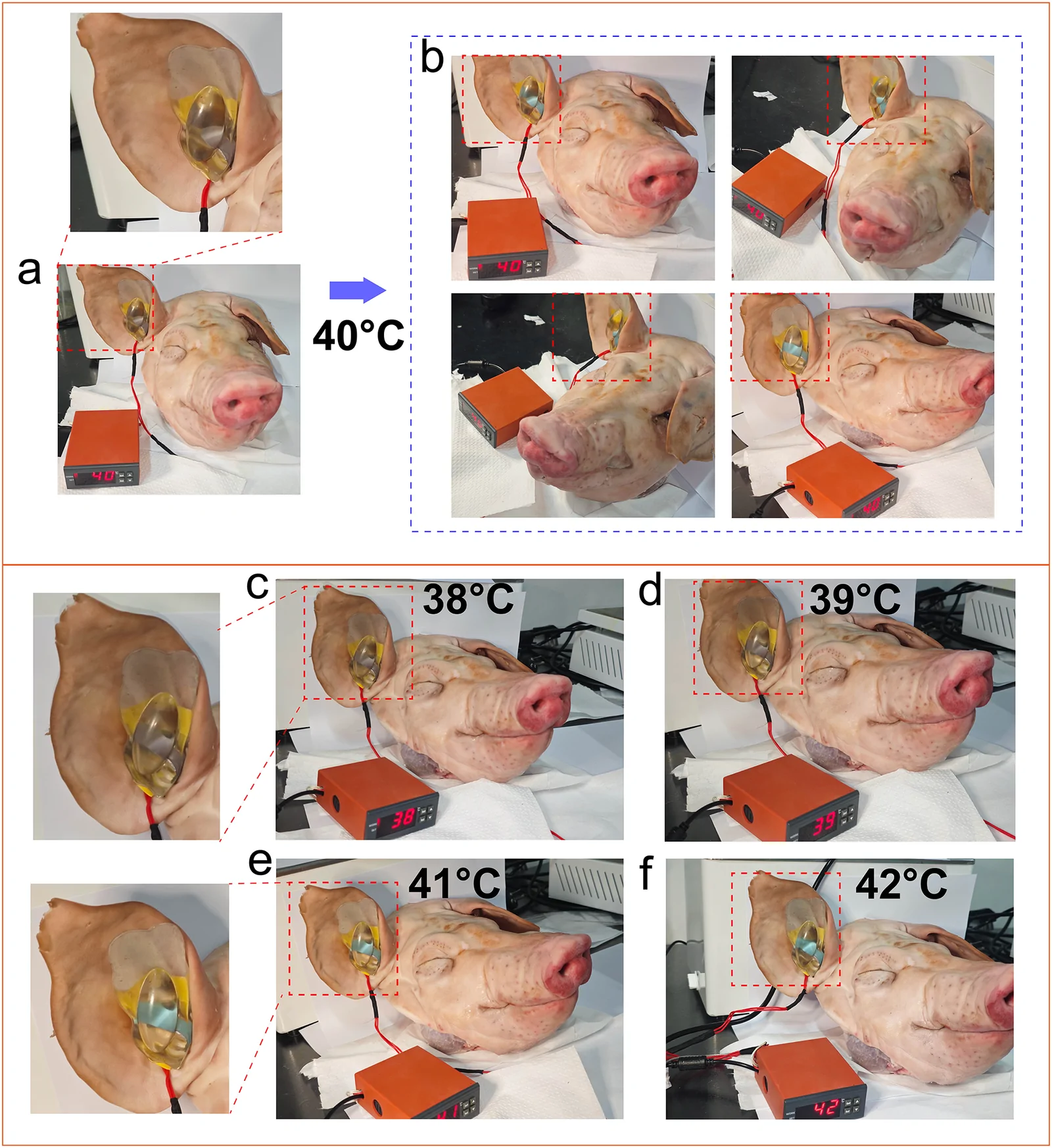
The simulation experiment with earplugs on the pig’s ears: (a) The initial photo at 40 °C; (b) The photos taken from different angles after heating at 40 °C for 5 min, (c) The photo after heating at 38 °C for 5 min; (d) The photo after heating at 39 °C for 5 min; (e) The photo after heating at 41 °C for 5 min; (f) The photo after heating at 42 °C for 5 min.

To evaluate the practical application potential of the thermochromic coating, earplugs were fabricated by wrapping aluminum strips coated with the thermochromic layer in silicone rubber via 3D printing, and then inserted into pig ears. Corresponding heating plates were also constructed to perform simulation experiments. Since the PU/TM/HBN-12/BD-0.24 coating exhibited the best overall performance, it was selected for this study. As shown in Fig 11a, b, at 40 °C the earplugs initially appeared light purple, which was not clearly distinguishable from the color of pig ear skin. After approximately 60 s, the earplugs transitioned to a bright blue color, which showed a clear contrast with the pig ear skin. In addition, color transition behavior was evaluated at 38, 39, 41, and 42 °C, as shown in Fig 11c-f. At 38 and 39 °C, the earplugs remained purple after 5 min of exposure, whereas at 41 and 42 °C, the rapidly changed to a bright blue color. These results indicate that the thermochromic coating and the fabricated earplugs can effectively reflect the critical temperature associated with fever in pigs, demonstrating potential for practical application.

## 4. Conclusion

In conclusion, an earplug integrated with a thermochromic coating was successfully fabricated. By systematically tuning the filler content, coating thickness, and dye concentration, the coating exhibited a distinct color transition even when positioned away from the heat source. Simulation results further indicate that, under a 40 °C external heat source, the effective color‑transition temperature of the coating within the earplug should be maintained at approximately 34 °C. Experimental results demonstrate that the incorporation of HBNs significantly enhances the color-transition speed, increases the color-transition distance, and narrows the color-transition temperature range, primarily due to the improved thermal conductivity. However, a high HBNs content of 12 wt.% reduces the ΔE color difference. Increasing coating thickness prolongs the complete color-transition time, while the 30 μm sample exhibits the highest ΔE value. The incorporation of blue dye enables the coating to present clearly distinguishable color states before and after thermochromic transition. The ΔEvalue between the pre-transition and post-transition states decreases with increasing blue dye concentration, whereas the $\Delta E^{*}$ value between the post-transition state and pig skin color increases with increasing dye concentration. At 40 °C, the thermochromic earplugs rapidly transition from light purple to blue, suggesting potential application as a visual indicator for fever detection in pigs under controlled conditions. The pig ear validation experiment in this article currently only used one sample (n = 1), and the preliminary results show a significant color change after coating, which provides a useful direction for our future research. Next, we will increase the number of repeated samples to improve the statistical reliability of the conclusions.

## Supporting information

S1 Raw data Simulation results for earplugs with a heat source temperature of 38 °C. (DOCX)

S2 Raw data Simulation results for earplugs with a heat source temperature of 40 °C. (DOCX)

S3 Raw data The raw data of XRD. (XLSX)

S4 Raw data The raw data of FTIR. (XLSX)

S5 Raw data Heating-cooling cycle tests. (XLSX)

S6 Raw data The color difference calculated based on the skin color of pig’s head. (XLSX)

S7 Raw data The color difference values at 33–37 °C. (XLSX)

S8 Raw data The color difference values of samples with different HBN contents change over time. (XLSX)

S9 Raw data The variation in color difference of samples of different thicknesses over time. (XLSX)

S10 Raw data The variation in color difference of samples with different pigment concentrations over time. (XLSX)

S11 Raw data Thermal Conductivity. (XLSX)

S12 Raw data T-t curve data for PU-TM-BD-2.4 with different thickness. (XLSX)

S13 Raw data T-t curve data for PU-TM-HBN-x. (XLSX)
