# Supplementary material for: Effects of coating composition and thickness on thermochromic performance and application in earplugs for swine fever
Source: PLoS One. 2026 Sep 22;21(9):e0358752. doi: 10.1371/journal.pone.0358752 (PMC13596828; doi:10.1371/journal.pone.0358752)
Supplement: Supplementary file 1 [file pone.0358752.s001.docx]

<https://www.comsol.com/>

Heating at 38°C

| Report date | Jun 18, 2026, 10:31:39 PM |
| --- | --- |

Contents

**1.	Global Definitions**

**1.1.	Shared Properties**

**2.	Component**

**2.1.	Definitions**

**2.2.	Geometry**

**2.3.	Materials**

**2.4.	Solid Heat Transfer**

**2.5.	Mesh**

**3.	Study**

**3.1.	Transient**

**3.2.	Solver Configurations**

**4.	Results**

**4.1.	Datasets**

**4.2.	Derived Values**

**4.3.	Tables**

**4.4.	Plot Groups**

# 1 Global Definitions

| Date | Feb 1, 2026, 3:56:45 PM |
| --- | --- |

Global settings

| Name | Heating at 38°C.mph |
| --- | --- |
| Version | COMSOL Multiphysics 6.3 (Build: 290) |
| Unit system | SI |

Used products

Design Module

COMSOL Multiphysics

CAD Import Module

Computer information

| CPU | Intel64 Family 6 Model 183 Stepping 1, 16 cores, 31.63 GB RAM |
| --- | --- |
| Operating system | Windows 11 |

## 1.1 Shared Properties

### 1.1.1 Default Model Input

| Tag | cminpt |
| --- | --- |

# 2 Component

| Date | Oct 19, 2025, 3:09:49 PM |
| --- | --- |

Settings

| **Description** | **Value** |
| --- | --- |
| Unit system | Same as global system (SI) |
| Geometry shape function | Automatic |

Spatial frame coordinates

| **First** | **Second** | **Third** |
| --- | --- | --- |
| x | y | z |

Material frame coordinates

| **First** | **Second** | **Third** |
| --- | --- | --- |
| X | Y | Z |

Geometry frame coordinates

| **First** | **Second** | **Third** |
| --- | --- | --- |
| Xg | Yg | Zg |

Mesh frame coordinates

| **First** | **Second** | **Third** |
| --- | --- | --- |
| Xm | Ym | Zm |

## 2.1 Definitions

### 2.1.1 Probes

| Probe type | Boundary probe |
| --- | --- |

Selection

| Geometric entity level | Boundary |
| --- | --- |
| Selection | Geometry geom1: Dimension 2: Boundaries 108–109, 121, 146, 156 |

Selection

Expression

| **Description** | **Value** |
| --- | --- |
| Expression | T |
| Table and plot unit | °C |

Table and window settings

| **Description** | **Value** |
| --- | --- |
| Plot window |  |

### 2.1.2 Coordinate Systems

| Coordinate system type | Boundary system |
| --- | --- |
| Tag | sys1 |

Coordinate names

| **First** | **Second** | **Third** |
| --- | --- | --- |
| t1 | t2 | n |

## 2.2 Geometry

Units

| Length unit | m |
| --- | --- |
| Angular unit | deg |

Geometry statistics

| **Description** | **Value** |
| --- | --- |
| Space dimension | 3 |
| Number of domains | 4 |
| Number of boundaries | 354 |
| Number of edges | 918 |
| Number of vertices | 568 |

## 2.3 Materials

### 2.3.1 Aluminum

Aluminum

Selection

| Geometric entity level | Domain |
| --- | --- |
| Selection | Geometry geom1: Dimension 3: Domains 2–4 |

Material parameters

| **Name** | **Value** | **Unit** | **Property group** |
| --- | --- | --- | --- |
| Heat capacity at constant pressure | 900 | J/(kg·K) | Basic |
| Thermal conductivity | 238 | W/(m·K) | Basic |
| Density | 2700 | kg/m³ | Basic |

Basic

| **Description** | **Value** | **Unit** |
| --- | --- | --- |
| Heat capacity at constant pressure | 900 | J/(kg·K) |
| Thermal conductivity | 238 | W/(m·K) |
| Density | 2700 | kg/m³ |

### 2.3.2 Rubber

Rubber

Selection

| Geometric entity level | Domain |
| --- | --- |
| Selection | Geometry geom1: Dimension 3: Domain 1 |

Material parameters

| **Name** | **Value** | **Unit** | **Property group** |
| --- | --- | --- | --- |
| Density | 1100 | kg/m³ | Basic |
| Thermal conductivity | 0.5 | W/(m·K) | Basic |
| Heat capacity at constant pressure | 1900 | J/(kg·K) | Basic |

Basic

| **Description** | **Value** | **Unit** |
| --- | --- | --- |
| Density | 1100 | kg/m³ |
| Thermal conductivity | 0.5 | W/(m·K) |
| Heat capacity at constant pressure | 1900 | J/(kg·K) |

## 2.4 Solid Heat Transfer

Used products

COMSOL Multiphysics

Selection

| Geometric entity level | Domain |
| --- | --- |
| Selection | Geometry geom1: Dimension 3: All domains |

Equations

### 2.4.1 Interface Settings

#### Discretization

Settings

| **Description** | **Value** |
| --- | --- |
| Temperature | Quadratic Lagrange |

Settings

| **Description** | **Value** |
| --- | --- |
| Equation form | Study controlled |

#### Physical Model

Settings

| **Description** | **Value** | **Unit** |
| --- | --- | --- |
| Reference temperature | User defined |  |
| Reference temperature | 293.15 | K |

### 2.4.2 Variables

| **Name** | **Expression** | **Unit** | **Description** | **Selection** | **Details** |
| --- | --- | --- | --- | --- | --- |
| ht.Tref | model.input.Tref | K | Reference temperature | Global | Meta |
| ht.C_effExt | 0 | J/(m³·K) | Effective volumetric heat capacity | Domains 1–4 | + operation |
| ht.EMatExt | 0 | Pa | Young's modulus | Domains 1–4 | + operation |
| ht.Tvar | T | K | Temperature | Domains 1–4 |  |
| ht.Tvar | T | K | Temperature | Boundaries 1–354 |  |
| ht.Tvar | T | K | Temperature | Edges 1–918 |  |
| ht.Tvar | T | K | Temperature | Points 1–568 |  |
| ht.nknExt | 0 | W/(m·K) | Help variable | Domains 1–4 | + operation |
| ht.TextFace | 0 | K | External temperature | Domains 1–4 | + operation |
| ht.nuMatExt | 0 | 1 | Poisson's ratio | Domains 1–4 | + operation |
| ht.d | 1 | 1 | Thickness | Domains 1–4 |  |
| ht.chiT | 0 | 1/Pa | Isothermal compressibility coefficient | Domains 1–4 |  |
| ht.HRef | 0 | J/kg | Reference enthalpy | Domains 1–4 |  |
| ht.alphap | 0 | 1/K | Isobaric compressibility coefficient | Domains 1–4 |  |
| ht.DeltaH | ht.DeltaH_add | J/kg | Sensible enthalpy | Domains 1–4 |  |
| ht.DeltaH_cst | ht.DeltaH_add_cst | J/kg | Sensible enthalpy, constant material properties | Domains 1–4 |  |
| ht.DeltaH_add | 0 | J/kg | Sensible enthalpy | Domains 1–4 | + operation |
| ht.DeltaH_add_cst | 0 | J/kg | Sensible enthalpy, constant material properties | Domains 1–4 | + operation |
| .... | .... | .... | .... | .... | .... |
| ht.varIntSpa | ht.d | 1 | Intermediate variable | Domains 1–4 | Meta |

### 2.4.3 Solid

Selection

| Geometric entity level | Domain |
| --- | --- |
| Selection | Geometry geom1: Dimension 3: All domains |

Equations

#### Heat Conduction, Solid

Settings

| **Description** | **Value** |
| --- | --- |
| Thermal conductivity | From material |

#### Thermodynamics, Solid

Settings

| **Description** | **Value** |
| --- | --- |
| Density | From material |
| Heat capacity at constant pressure | From material |

#### Coordinate System Selection

Settings

| **Description** | **Value** |
| --- | --- |
| Coordinate system | Global coordinate system |

Properties from material

| **Property** | **Material** | **Property group** |
| --- | --- | --- |
| Thermal conductivity | Aluminum | Basic |
| Density | Aluminum | Basic |
| Heat capacity at constant pressure | Aluminum | Basic |
| Thermal conductivity | Rubber | Basic |
| Density | Rubber | Basic |
| Heat capacity at constant pressure | Rubber | Basic |

#### Variables

| **Name** | **Expression** | **Unit** | **Description** | **Selection** | **Details** |
| --- | --- | --- | --- | --- | --- |
| domflux.Tx | ht.dfluxx*ht.d | W/m² | Domain flux, x-component | Domains 1–4 |  |
| domflux.Ty | ht.dfluxy*ht.d | W/m² | Domain flux, y-component | Domains 1–4 |  |
| domflux.Tz | ht.dfluxz*ht.d | W/m² | Domain flux, z-component | Domains 1–4 |  |
| ht.chiT | 0 | 1/Pa | Isothermal compressibility coefficient | Domains 1–4 |  |
| ht.alphap | -d(ht.rho,T)/max(ht.rho,eps) | 1/K | Isobaric compressibility coefficient | Domains 1–4 |  |
| ht.DeltaH_add | integrate(ht.CpInt,ht.TInt,ht.DeltaH_Tlow,T) | J/kg | Sensible enthalpy | Domains 1–4 | + operation |
| .... | .... | .... | .... | .... | .... |
| ht.Cp_ref | subst(material.Cp,ht.solid1.minput_pressure,ht.pref,ht.solid1.minput_temperature,ht.Tref) | J/(kg·K) | Reference heat capacity | Domains 1–4 | Meta |

#### Shape functions

| **Name** | **Shape function** | **Unit** | **Description** | **Shape frame** | **Selection** |
| --- | --- | --- | --- | --- | --- |
| T | Lagrange (Quadratic) | K | Temperature | Material | Domains 1–4 |
| T | Lagrange (Quadratic) | K | Temperature | Spatial | Domains 1–4 |
| T | Lagrange (Quadratic) | K | Temperature | Geometry | Domains 1–4 |
| T | Lagrange (Quadratic) | K | Temperature | Mesh | Domains 1–4 |

#### Weak Expressions

| **Weak expression** | **Integration order** | **Integration frame** | **Selection** |
| --- | --- | --- | --- |
| (ht.dfluxx*test(Tx)+ht.dfluxy*test(Ty)+ht.dfluxz*test(Tz))*ht.d | 4 | Spatial | Domains 1–4 |
| -ht.C_eff*ht.timeDerivative*test(T)*ht.d | 4 | Spatial | Domains 1–4 |

### 2.4.4 Initial Values

Selection

| Geometric entity level | Domain |
| --- | --- |
| Selection | Geometry geom1: Dimension 3: All domains |

#### Initial Values

Settings

| **Description** | **Value** | **Unit** |
| --- | --- | --- |
| Temperature | User defined |  |
| Temperature | 293.15 | K |

#### Variables

| **Name** | **Expression** | **Unit** | **Description** | **Selection** |
| --- | --- | --- | --- | --- |
| ht.Tinit | 293.15[K] | K | Initial temperature | Domains 1–4 |

### 2.4.5 Thermal Insulation

Selection

| Geometric entity level | Boundary |
| --- | --- |
| Selection | Geometry geom1: Dimension 2: All boundaries |

Equations

#### Variables

| **Name** | **Expression** | **Unit** | **Description** | **Selection** |
| --- | --- | --- | --- | --- |
| ht.ins1.ntfluxInt | ht.ins1.intExtBnd(ht.ntflux*ht.varIntSpa)+ht.ins1.intIntBnd(ht.ncflux_u*up(ht.varIntSpa)+ht.ncflux_d*down(ht.varIntSpa)) | W | Total net heat rate | Global |
| ht.ins1.ntfluxInt_cst | ht.ins1.intExtBnd(ht.ntflux*ht.varIntSpa)+ht.ins1.intIntBnd(ht.ncflux_u*up(ht.varIntSpa)+ht.ncflux_d*down(ht.varIntSpa)) | W | Total net heat rate, constant material properties | Global |
| ht.ins1.ntefluxInt | ht.ins1.intExtBnd(ht.nteflux*ht.varIntSpa)+ht.ins1.intIntBnd(ht.nthflux_u*up(ht.varIntSpa)+ht.nthflux_d*down(ht.varIntSpa)) | W | Total net energy rate | Global |
| ht.ins1.ntefluxInt_cst | ht.ins1.intExtBnd(ht.nteflux_cst*ht.varIntSpa)+ht.ins1.intIntBnd(ht.nthflux_cst_u*up(ht.varIntSpa)+ht.nthflux_cst_d*down(ht.varIntSpa)) | W | Total net energy rate, constant material properties | Global |
| .... | .... | .... | .... | .... |
| ht.ins1.Tave | nojac(ht.ins1.intBnd(ht.varIntSpa*ht.rho*ht.Cp*T*max(abs(ht.ux*ht.nxmesh+ht.uy*ht.nymesh+ht.uz*ht.nzmesh),eps)))/nojac(ht.ins1.intBnd(ht.varIntSpa*ht.rho*ht.Cp*max(abs(ht.ux*ht.nxmesh+ht.uy*ht.nymesh+ht.uz*ht.nzmesh),eps))) | K | Weighted average temperature | Global |

#### Shape functions

| **Name** | **Shape function** | **Unit** | **Description** | **Shape frame** | **Selection** | **Details** |
| --- | --- | --- | --- | --- | --- | --- |
| T | Lagrange (Quadratic) | K | Temperature | Spatial | No boundaries | Slit |
| T | Lagrange (Quadratic) | K | Temperature | Material | No boundaries | Slit |
| T | Lagrange (Quadratic) | K | Temperature | Geometry | No boundaries | Slit |
| T | Lagrange (Quadratic) | K | Temperature | Mesh | No boundaries | Slit |

### 2.4.6 Heat Source

Selection

| Geometric entity level | Domain |
| --- | --- |
| Selection | Geometry geom1: Dimension 3: Domains 2–4 |

Equations

#### Heat Source

Settings

| **Description** | **Value** | **Unit** |
| --- | --- | --- |
| Heat source | General source |  |
| Heat source | User defined |  |
| Heat source | 400 | W/m³ |

#### Variables

| **Name** | **Expression** | **Unit** | **Description** | **Selection** | **Details** |
| --- | --- | --- | --- | --- | --- |
| ht.Q | ht.hs1.Q*spatial.detInvF | W/m³ | Heat source | Domains 2–4 | + operation |
| ht.Qtot | ht.hs1.Q*spatial.detInvF | W/m³ | Total heat source | Domains 2–4 | + operation |
| ht.hs1.Q0 | 400 | W/m³ | Heat source | Domains 2–4 |  |
| ht.hs1.Q | ht.hs1.Q0 | W/m³ | Heat source | Domains 2–4 |  |

#### Weak Expressions

| **Weak expression** | **Integration order** | **Integration frame** | **Selection** |
| --- | --- | --- | --- |
| ht.hs1.Q*test(T)*ht.d | 4 | Material | Domains 2–4 |

### 2.4.7 Surface-to-Ambient Radiation

Selection

| Geometric entity level | Boundary |
| --- | --- |
| Selection | Geometry geom1: Dimension 2: Boundaries 1–35, 38–60, 62–65, 67–106, 113, 116, 124–126, 149–150, 157, 160, 172–174, 177–178, 199, 227, 261, 265, 275, 278, 284, 288, 292–294, 300–301, 305–307, 311–312, 314–316, 321–322, 326, 330–354 |

Equations

#### Surface-to-Ambient Radiation

Settings

| **Description** | **Value** | **Unit** |
| --- | --- | --- |
| Surface emissivity | User defined |  |
| Surface emissivity | 0.95 | 1 |
| Ambient temperature | User defined |  |
| Ambient temperature | 293.15 | K |

#### Variables

| **Name** | **Expression** | **Unit** | **Description** | **Selection** | **Details** |
| --- | --- | --- | --- | --- | --- |
| ht.rflux | ht.sar1.rflux | W/m² | Radiative heat flux | Boundaries 1–35, 38–60, 62–65, 67–106, 113, 116, 124–126, 149–150, 157, 160, 172–174, 177–178, 199, 227, 261, 265, 275, 278, 284, 288, 292–294, 300–301, 305–307, 311–312, 314–316, 321–322, 326, 330–354 | + operation |
| ht.epsilon_rad | 0.95 | 1 | Surface emissivity | Boundaries 1–35, 38–60, 62–65, 67–106, 113, 116, 124–126, 149–150, 157, 160, 172–174, 177–178, 199, 227, 261, 265, 275, 278, 284, 288, 292–294, 300–301, 305–307, 311–312, 314–316, 321–322, 326, 330–354 |  |
| ht.Tamb | 293.15[K] | K | Ambient temperature | Boundaries 1–35, 38–60, 62–65, 67–106, 113, 116, 124–126, 149–150, 157, 160, 172–174, 177–178, 199, 227, 261, 265, 275, 278, 284, 288, 292–294, 300–301, 305–307, 311–312, 314–316, 321–322, 326, 330–354 |  |
| ht.sar1.Tvar | ht.Tu | K | Temperature | Boundaries 1–35, 38–60, 62–65, 67–106, 113, 116, 124–126, 149–150, 157, 160, 172–174, 177–178, 199, 227, 261, 265, 275, 278, 284, 288, 292–294, 300–301, 305–307, 311–312, 314–316, 321–322, 326, 330–354 |  |
| ht.sar1.rflux | ht.epsilon_rad*sigma_const*((ht.Tamb-0[K])^4-(ht.sar1.Tvar-0[K])^4) | W/m² | Radiative heat flux | Boundaries 1–35, 38–60, 62–65, 67–106, 113, 116, 124–126, 149–150, 157, 160, 172–174, 177–178, 199, 227, 261, 265, 275, 278, 284, 288, 292–294, 300–301, 305–307, 311–312, 314–316, 321–322, 326, 330–354 |  |
| ht.sar1.ntfluxInt | ht.sar1.intExtBnd(ht.ntflux*ht.varIntSpa)+ht.sar1.intIntBnd(ht.ncflux_u*up(ht.varIntSpa)+ht.ncflux_d*down(ht.varIntSpa)) | W | Total net heat rate | Global |  |
| ht.sar1.ntfluxInt_cst | ht.sar1.intExtBnd(ht.ntflux*ht.varIntSpa)+ht.sar1.intIntBnd(ht.ncflux_u*up(ht.varIntSpa)+ht.ncflux_d*down(ht.varIntSpa)) | W | Total net heat rate, constant material properties | Global |  |
| ht.sar1.ntefluxInt | ht.sar1.intExtBnd(ht.nteflux*ht.varIntSpa)+ht.sar1.intIntBnd(ht.nthflux_u*up(ht.varIntSpa)+ht.nthflux_d*down(ht.varIntSpa)) | W | Total net energy rate | Global |  |
| ht.sar1.ntefluxInt_cst | ht.sar1.intExtBnd(ht.nteflux_cst*ht.varIntSpa)+ht.sar1.intIntBnd(ht.nthflux_cst_u*up(ht.varIntSpa)+ht.nthflux_cst_d*down(ht.varIntSpa)) | W | Total net energy rate, constant material properties | Global |  |
| ht.sar1.ntfluxInt_u | ht.sar1.intIntBnd(ht.ntflux_u*up(ht.varIntSpa)) | W | Total net heat rate, upside | Global |  |
| ht.sar1.ntfluxInt_cst_u | ht.sar1.intIntBnd(ht.ntflux_u*up(ht.varIntSpa)) | W | Total net heat rate, constant material properties, upside | Global |  |
| ht.sar1.ntefluxInt_u | ht.sar1.intIntBnd(ht.nteflux_u*up(ht.varIntSpa)) | W | Total net energy rate, upside | Global |  |
| ht.sar1.ntefluxInt_cst_u | ht.sar1.intIntBnd(ht.nteflux_cst_u*up(ht.varIntSpa)) | W | Total net energy rate, constant material properties, upside | Global |  |
| ht.sar1.ntfluxInt_d | ht.sar1.intIntBnd(ht.ntflux_d*down(ht.varIntSpa)) | W | Total net heat rate, downside | Global |  |
| ht.sar1.ntfluxInt_cst_d | ht.sar1.intIntBnd(ht.ntflux_d*down(ht.varIntSpa)) | W | Total net heat rate, constant material properties, downside | Global |  |
| ht.sar1.ntefluxInt_d | ht.sar1.intIntBnd(ht.nteflux_d*down(ht.varIntSpa)) | W | Total net energy rate, downside | Global |  |
| ht.sar1.ntefluxInt_cst_d | ht.sar1.intIntBnd(ht.nteflux_cst_d*down(ht.varIntSpa)) | W | Total net energy rate, constant material properties, downside | Global |  |
| ht.sar1.Tave | nojac(ht.sar1.intBnd(ht.varIntSpa*ht.rho*ht.Cp*T*max(abs(ht.ux*ht.nxmesh+ht.uy*ht.nymesh+ht.uz*ht.nzmesh),eps)))/nojac(ht.sar1.intBnd(ht.varIntSpa*ht.rho*ht.Cp*max(abs(ht.ux*ht.nxmesh+ht.uy*ht.nymesh+ht.uz*ht.nzmesh),eps))) | K | Weighted average temperature | Global |  |

#### Weak Expressions

| **Weak expression** | **Integration order** | **Integration frame** | **Selection** |
| --- | --- | --- | --- |
| ht.sar1.rflux*test(ht.sar1.Tvar)*ht.d | 4 | Spatial | Boundaries 1–35, 38–60, 62–65, 67–106, 113, 116, 124–126, 149–150, 157, 160, 172–174, 177–178, 199, 227, 261, 265, 275, 278, 284, 288, 292–294, 300–301, 305–307, 311–312, 314–316, 321–322, 326, 330–354 |

## 2.5 Mesh

Mesh statistics

| **Description** | **Value** |
| --- | --- |
| Status | Complete mesh |
| Mesh vertices | 11562 |
| Tetrahedra | 47807 |
| Triangles | 16570 |
| Edge elements | 2669 |
| Vertex elements | 568 |
| Number of elements | 47807 |
| Minimum element quality | 0.02022 |
| Average element quality | 0.6008 |
| Element volume ratio | 4.2203E-8 |
| Mesh volume | 4.857E-5 m³ |

### 2.5.1 Size1

Settings

| **Description** | **Value** |
| --- | --- |
| Maximum element size | 0.04449 |
| Minimum element size | 0.001606 |
| Resolution of narrow regions | 0.2 |
| Maximum element growth rate | 1.75 |
| Predefined size | Extremely coarse |
| Custom element size | Custom |

### 2.5.2 Size2

Selection

| Geometric entity level | Boundary |
| --- | --- |
| Selection | Geometry geom1: Dimension 2: Boundaries 1–354 |

Settings

| **Description** | **Value** |
| --- | --- |
| Maximum element size | 0.01017 |
| Minimum element size | 3.051E-4 |
| Curvature factor | Off |
| Resolution of narrow regions | 0.1 |
| Resolution of narrow regions | Off |
| Maximum element growth rate | 2 |
| Maximum element growth rate | Off |
| Predefined size | Extremely coarse |
| Custom element size | Custom |

### 2.5.3 Ftet1

Selection

| Geometric entity level | Domain |
| --- | --- |
| Selection | Remaining |

Settings

| **Description** | **Value** |
| --- | --- |
| Avoid inverted curved elements | On |

Information

| **Description** | **Value** |
| --- | --- |
| Last build time | 4 seconds |
| Built with | COMSOL 6.3.0.290 (win64), Jun 3, 2026, 9:28:24 PM |

# 3 Study

Computation information

| Computation time | 2 min 34 s |
| --- | --- |

## 3.1 Solver Configurations

### 3.1.1 A

#### Compile Equations

Study and step

| **Description** | **Value** |
| --- | --- |
| Use study |  |
| Use study step | ST |

#### v1

General

| **Description** | **Value** |
| --- | --- |
| Defined by study step |  |

Initial value calculation constants

| **Constant name** | **Initial-value source** |
| --- | --- |
| t | {range(0, 0.1, 120)}[s] |
| timestep | 0.12[s] |

Log

##### comp1.T

General

| **Description** | **Value** |
| --- | --- |
| Field components | comp1.T |
| Internal variables | {comp1.ht.dt2Inv_T, comp1.uflux.T, comp1.dflux.T} |

#### t1

General

| **Description** | **Value** |
| --- | --- |
| Defined by study step |  |
| Output times | {0, 0.1, 0.2, 0.3, 0.4, 0.5, 0.6, 0.7, 0.8, 0.9, 1, 1.1, 1.2, 1.3, 1.4, 1.5, 1.6, 1.7, 1.8, 1.9, 2, 2.1, 2.2, 2.3, 2.4, 2.5, 2.6, 2.7, 2.8, 2.9, 3, 3.1, 3.2, 3.3, 3.4, 3.5, 3.6, 3.7, 3.8, 3.9, 4, 4.1, 4.2, 4.3, 4.4, 4.5, 4.6, 4.7, 4.8, 4.9, 5, 5.1, 5.2, 5.3, 5.4, 5.5, 5.6, 5.7, 5.8, 5.9, 6, 6.1, 6.2, 6.3, 6.4, 6.5, 6.6, 6.7, 6.8, 6.9, 7, 7.1, 7.2, 7.3, 7.4, 7.5, 7.6, 7.7, 7.8, 7.9, 8, 8.1, 8.2, 8.3, 8.4, 8.5, 8.6, 8.7, 8.8, 8.9, 9, 9.1, 9.2, 9.3, 9.4, 9.5, 9.6, 9.7, 9.8, 9.9, 10, 10.1, 10.2, 10.3, 10.4, 10.5, 10.6, 10.7, 10.8, 10.9, 11, 11.1, 11.2, 11.3, 11.4, 11.5, 11.6, 11.7, 11.8, 11.9, 12, 12.1, 12.2, 12.3, 12.4, 12.5, 12.6, 12.7, 12.8, 12.9, 13, 13.1, 13.2, 13.3, 13.4, 13.5, 13.6, 13.7, 13.8, 13.9, 14, 14.1, 14.2, 14.3, 14.4, 14.5, 14.6, 14.7, 14.8, 14.9, 15, 15.1, 15.2, 15.3, 15.4, 15.5, 15.6, 15.7, 15.8, 15.9, 16, 16.1, 16.2, 16.3, 16.4, 16.5, 16.6, 16.7, 16.8, 16.9, 17, 17.1, 17.2, 17.3, 17.4, 17.5, 17.6, 17.7, 17.8, 17.9, 18, 18.1, 18.2, 18.3, 18.4, 18.5, 18.6, 18.7, 18.8, 18.9, 19, 19.1, 19.2, 19.3, 19.4, 19.5, 19.6, 19.7, 19.8, 19.9, 20, 20.1, 20.2, 20.3, 20.4, 20.5, 20.6, 20.7, 20.8, 20.9, 21, 21.1, 21.2, 21.3, 21.4, 21.5, 21.6, 21.7, 21.8, 21.9, 22, 22.1, 22.2, 22.3, 22.4, 22.5, 22.6, 22.7, 22.8, 22.9, 23, 23.1, 23.2, 23.3, 23.4, 23.5, 23.6, 23.7, 23.8, 23.9, 24, 24.1, 24.2, 24.3, 24.4, 24.5, 24.6, 24.7, 24.8, 24.9, 25, 25.1, 25.2, 25.3, 25.4, 25.5, 25.6, 25.7, 25.8, 25.9, 26, 26.1, 26.2, 26.3, 26.4, 26.5, 26.6, 26.7, 26.8, 26.9, 27, 27.1, 27.2, 27.3, 27.4, 27.5, 27.6, 27.7, 27.8, 27.9, 28, 28.1, 28.2, 28.3, 28.4, 28.5, 28.6, 28.7, 28.8, 28.9, 29, 29.1, 29.2, 29.3, 29.4, 29.5, 29.6, 29.7, 29.8, 29.9, 30, 30.1, 30.2, 30.3, 30.4, 30.5, 30.6, 30.7, 30.8, 30.9, 31, 31.1, 31.2, 31.3, 31.4, 31.5, 31.6, 31.7, 31.8, 31.9, 32, 32.1, 32.2, 32.3, 32.4, 32.5, 32.6, 32.7, 32.8, 32.9, 33, 33.1, 33.2, 33.3, 33.4, 33.5, 33.6, 33.7, 33.8, 33.9, 34, 34.1, 34.2, 34.3, 34.4, 34.5, 34.6, 34.7, 34.8, 34.9, 35, 35.1, 35.2, 35.3, 35.4, 35.5, 35.6, 35.7, 35.8, 35.9, 36, 36.1, 36.2, 36.3, 36.4, 36.5, 36.6, 36.7, 36.8, 36.9, 37, 37.1, 37.2, 37.3, 37.4, 37.5, 37.6, 37.7, 37.8, 37.9, 38, 38.1, 38.2, 38.3, 38.4, 38.5, 38.6, 38.7, 38.8, 38.9, 39, 39.1, 39.2, 39.3, 39.4, 39.5, 39.6, 39.7, 39.8, 39.9, 40, 40.1, 40.2, 40.3, 40.4, 40.5, 40.6, 40.7, 40.8, 40.9, 41, 41.1, 41.2, 41.3, 41.4, 41.5, 41.6, 41.7, 41.8, 41.9, 42, 42.1, 42.2, 42.3, 42.4, 42.5, 42.6, 42.7, 42.8, 42.9, 43, 43.1, 43.2, 43.3, 43.4, 43.5, 43.6, 43.7, 43.8, 43.9, 44, 44.1, 44.2, 44.3, 44.4, 44.5, 44.6, 44.7, 44.8, 44.9, 45, 45.1, 45.2, 45.3, 45.4, 45.5, 45.6, 45.7, 45.8, 45.9, 46, 46.1, 46.2, 46.3, 46.4, 46.5, 46.6, 46.7, 46.8, 46.9, 47, 47.1, 47.2, 47.3, 47.4, 47.5, 47.6, 47.7, 47.8, 47.9, 48, 48.1, 48.2, 48.3, 48.4, 48.5, 48.6, 48.7, 48.8, 48.9, 49, 49.1, 49.2, 49.3, 49.4, 49.5, 49.6, 49.7, 49.8, 49.9, 50, 50.1, 50.2, 50.3, 50.4, 50.5, 50.6, 50.7, 50.8, 50.9, 51, 51.1, 51.2, 51.3, 51.4, 51.5, 51.6, 51.7, 51.8, 51.9, 52, 52.1, 52.2, 52.3, 52.4, 52.5, 52.6, 52.7, 52.8, 52.9, 53, 53.1, 53.2, 53.3, 53.4, 53.5, 53.6, 53.7, 53.8, 53.9, 54, 54.1, 54.2, 54.3, 54.4, 54.5, 54.6, 54.7, 54.8, 54.9, 55, 55.1, 55.2, 55.3, 55.4, 55.5, 55.6, 55.7, 55.8, 55.9, 56, 56.1, 56.2, 56.3, 56.4, 56.5, 56.6, 56.7, 56.8, 56.9, 57, 57.1, 57.2, 57.3, 57.4, 57.5, 57.6, 57.7, 57.8, 57.9, 58, 58.1, 58.2, 58.3, 58.4, 58.5, 58.6, 58.7, 58.8, 58.9, 59, 59.1, 59.2, 59.3, 59.4, 59.5, 59.6, 59.7, 59.8, 59.9, 60, 60.1, 60.2, 60.3, 60.4, 60.5, 60.6, 60.7, 60.8, 60.9, 61, 61.1, 61.2, 61.3, 61.4, 61.5, 61.6, 61.7, 61.8, 61.9, 62, 62.1, 62.2, 62.3, 62.4, 62.5, 62.6, 62.7, 62.8, 62.9, 63, 63.1, 63.2, 63.3, 63.4, 63.5, 63.6, 63.7, 63.8, 63.9, 64, 64.1, 64.2, 64.3, 64.4, 64.5, 64.6, 64.7, 64.8, 64.9, 65, 65.1, 65.2, 65.3, 65.4, 65.5, 65.6, 65.7, 65.8, 65.9, 66, 66.1, 66.2, 66.3, 66.4, 66.5, 66.6, 66.7, 66.8, 66.9, 67, 67.1, 67.2, 67.3, 67.4, 67.5, 67.6, 67.7, 67.8, 67.9, 68, 68.1, 68.2, 68.3, 68.4, 68.5, 68.6, 68.7, 68.8, 68.9, 69, 69.1, 69.2, 69.3, 69.4, 69.5, 69.6, 69.7, 69.8, 69.9, 70, 70.1, 70.2, 70.3, 70.4, 70.5, 70.6, 70.7, 70.8, 70.9, 71, 71.1, 71.2, 71.3, 71.4, 71.5, 71.6, 71.7, 71.8, 71.9, 72, 72.1, 72.2, 72.3, 72.4, 72.5, 72.6, 72.7, 72.8, 72.9, 73, 73.1, 73.2, 73.3, 73.4, 73.5, 73.6, 73.7, 73.8, 73.9, 74, 74.1, 74.2, 74.3, 74.4, 74.5, 74.6, 74.7, 74.8, 74.9, 75, 75.1, 75.2, 75.3, 75.4, 75.5, 75.6, 75.7, 75.8, 75.9, 76, 76.1, 76.2, 76.3, 76.4, 76.5, 76.6, 76.7, 76.8, 76.9, 77, 77.1, 77.2, 77.3, 77.4, 77.5, 77.6, 77.7, 77.8, 77.9, 78, 78.1, 78.2, 78.3, 78.4, 78.5, 78.6, 78.7, 78.8, 78.9, 79, 79.1, 79.2, 79.3, 79.4, 79.5, 79.6, 79.7, 79.8, 79.9, 80, 80.1, 80.2, 80.3, 80.4, 80.5, 80.6, 80.7, 80.8, 80.9, 81, 81.1, 81.2, 81.3, 81.4, 81.5, 81.6, 81.7, 81.8, 81.9, 82, 82.1, 82.2, 82.3, 82.4, 82.5, 82.6, 82.7, 82.8, 82.9, 83, 83.1, 83.2, 83.3, 83.4, 83.5, 83.6, 83.7, 83.8, 83.9, 84, 84.1, 84.2, 84.3, 84.4, 84.5, 84.6, 84.7, 84.8, 84.9, 85, 85.1, 85.2, 85.3, 85.4, 85.5, 85.6, 85.7, 85.8, 85.9, 86, 86.1, 86.2, 86.3, 86.4, 86.5, 86.6, 86.7, 86.8, 86.9, 87, 87.1, 87.2, 87.3, 87.4, 87.5, 87.6, 87.7, 87.8, 87.9, 88, 88.1, 88.2, 88.3, 88.4, 88.5, 88.6, 88.7, 88.8, 88.9, 89, 89.1, 89.2, 89.3, 89.4, 89.5, 89.6, 89.7, 89.8, 89.9, 90, 90.1, 90.2, 90.3, 90.4, 90.5, 90.6, 90.7, 90.8, 90.9, 91, 91.1, 91.2, 91.3, 91.4, 91.5, 91.6, 91.7, 91.8, 91.9, 92, 92.1, 92.2, 92.3, 92.4, 92.5, 92.6, 92.7, 92.8, 92.9, 93, 93.1, 93.2, 93.3, 93.4, 93.5, 93.6, 93.7, 93.8, 93.9, 94, 94.1, 94.2, 94.3, 94.4, 94.5, 94.6, 94.7, 94.8, 94.9, 95, 95.1, 95.2, 95.3, 95.4, 95.5, 95.6, 95.7, 95.8, 95.9, 96, 96.1, 96.2, 96.3, 96.4, 96.5, 96.6, 96.7, 96.8, 96.9, 97, 97.1, 97.2, 97.3, 97.4, 97.5, 97.6, 97.7, 97.8, 97.9, 98, 98.1, 98.2, 98.3, 98.4, 98.5, 98.6, 98.7, 98.8, 98.9, 99, 99.1, 99.2, 99.3, 99.4, 99.5, 99.6, 99.7, 99.8, 99.9, 100, 100.1, 100.2, 100.3, 100.4, 100.5, 100.6, 100.7, 100.8, 100.9, 101, 101.1, 101.2, 101.3, 101.4, 101.5, 101.6, 101.7, 101.8, 101.9, 102, 102.1, 102.2, 102.3, 102.4, 102.5, 102.6, 102.7, 102.8, 102.9, 103, 103.1, 103.2, 103.3, 103.4, 103.5, 103.6, 103.7, 103.8, 103.9, 104, 104.1, 104.2, 104.3, 104.4, 104.5, 104.6, 104.7, 104.8, 104.9, 105, 105.1, 105.2, 105.3, 105.4, 105.5, 105.6, 105.7, 105.8, 105.9, 106, 106.1, 106.2, 106.3, 106.4, 106.5, 106.6, 106.7, 106.8, 106.9, 107, 107.1, 107.2, 107.3, 107.4, 107.5, 107.6, 107.7, 107.8, 107.9, 108, 108.1, 108.2, 108.3, 108.4, 108.5, 108.6, 108.7, 108.8, 108.9, 109, 109.1, 109.2, 109.3, 109.4, 109.5, 109.6, 109.7, 109.8, 109.9, 110, 110.1, 110.2, 110.3, 110.4, 110.5, 110.6, 110.7, 110.8, 110.9, 111, 111.1, 111.2, 111.3, 111.4, 111.5, 111.6, 111.7, 111.8, 111.9, 112, 112.1, 112.2, 112.3, 112.4, 112.5, 112.6, 112.7, 112.8, 112.9, 113, 113.1, 113.2, 113.3, 113.4, 113.5, 113.6, 113.7, 113.8, 113.9, 114, 114.1, 114.2, 114.3, 114.4, 114.5, 114.6, 114.7, 114.8, 114.9, 115, 115.1, 115.2, 115.3, 115.4, 115.5, 115.6, 115.7, 115.8, 115.9, 116, 116.1, 116.2, 116.3, 116.4, 116.5, 116.6, 116.7, 116.8, 116.9, 117, 117.1, 117.2, 117.3, 117.4, 117.5, 117.6, 117.7, 117.8, 117.9, 118, 118.1, 118.2, 118.3, 118.4, 118.5, 118.6, 118.7, 118.8, 118.9, 119, 119.1, 119.2, 119.3, 119.4, 119.5, 119.6, 119.7, 119.8, 119.9, 120} |

Absolute tolerance

| **Field** | **Method** | **Tolerance method** | **Tolerance factor** | **Derivative tolerance method** | **Time derivative factor** | **Tolerance** | **Tolerance for time derivatives** |
| --- | --- | --- | --- | --- | --- | --- | --- |
| T (comp1.T) | Use global | Factor | 0.1 | Automatic | 1 | 0.001 | 0.001 |

Time stepping

| **Description** | **Value** |
| --- | --- |
| Maximum BDF order | 2 |
| Error estimation | Exclude algebraic |

Log

```
   -        31.8           - out
   -        31.9           - out
   -          32           - out
   -        32.1           - out
   -        32.2           - out
   -        32.3           - out
   -        32.4           - out
   -        32.5           - out
   -        32.6           - out
   -        32.7           - out
   -        32.8           - out
   -        32.9           - out
   -          33           - out
   -        33.1           - out
   -        33.2           - out
   -        33.3           - out
   -        33.4           - out
   -        33.5           - out
   -        33.6           - out
   -        33.7           - out
   -        33.8           - out
   -        33.9           - out
   -          34           - out
   -        34.1           - out
   -        34.2           - out
   -        34.3           - out
   -        34.4           - out
   -        34.5           - out
   -        34.6           - out
   -        34.7           - out
   -        34.8           - out
   -        34.9           - out
   -          35           - out
   -        35.1           - out
   -        35.2           - out
   -        35.3           - out
   -        35.4           - out
   -        35.5           - out
   -        35.6           - out
   -        35.7           - out
   -        35.8           - out
   -        35.9           - out
   -          36           - out
   -        36.1           - out
   -        36.2           - out
   -        36.3           - out
   -        36.4           - out
   -        36.5           - out
   -        36.6           - out
   -        36.7           - out
   -        36.8           - out
   -        36.9           - out
   -          37           - out
   -        37.1           - out
   -        37.2           - out
   -        37.3           - out
   -        37.4           - out
   -        37.5           - out
   -        37.6           - out
   -        37.7           - out
   -        37.8           - out
   -        37.9           - out
   -          38           - out
   -        38.1           - out
   -        38.2           - out
   -        38.3           - out
   -        38.4           - out
   -        38.5           - out
   -        38.6           - out
   -        38.7           - out
   -        38.8           - out
   -        38.9           - out
   -          39           - out
   -        39.1           - out
   -        39.2           - out
   -        39.3           - out
  10       39.36          12       21   13   21     1     0      0    9e-14  1.1e-13
   -        39.4           - out
   -        39.5           - out
   -        39.6           - out
   -        39.7           - out
   -        39.8           - out
   -        39.9           - out
   -          40           - out
   -        40.1           - out
   -        40.2           - out
   -        40.3           - out
   -        40.4           - out
   -        40.5           - out
   -        40.6           - out
   -        40.7           - out
   -        40.8           - out
   -        40.9           - out
   -          41           - out
   -        41.1           - out
   -        41.2           - out
   -        41.3           - out
   -        41.4           - out
   -        41.5           - out
   -        41.6           - out
   -        41.7           - out
   -        41.8           - out
   -        41.9           - out
   -          42           - out
   -        42.1           - out
   -        42.2           - out
   -        42.3           - out
   -        42.4           - out
   -        42.5           - out
   -        42.6           - out
   -        42.7           - out
   -        42.8           - out
   -        42.9           - out
   -          43           - out
   -        43.1           - out
   -        43.2           - out
   -        43.3           - out
   -        43.4           - out
   -        43.5           - out
   -        43.6           - out
   -        43.7           - out
   -        43.8           - out
   -        43.9           - out
   -          44           - out
   -        44.1           - out
   -        44.2           - out
   -        44.3           - out
   -        44.4           - out
   -        44.5           - out
   -        44.6           - out
   -        44.7           - out
   -        44.8           - out
   -        44.9           - out
   -          45           - out
   -        45.1           - out
   -        45.2           - out
   -        45.3           - out
   -        45.4           - out
   -        45.5           - out
   -        45.6           - out
   -        45.7           - out
   -        45.8           - out
   -        45.9           - out
   -          46           - out
   -        46.1           - out
   -        46.2           - out
   -        46.3           - out
   -        46.4           - out
   -        46.5           - out
   -        46.6           - out
   -        46.7           - out
   -        46.8           - out
   -        46.9           - out
   -          47           - out
   -        47.1           - out
   -        47.2           - out
   -        47.3           - out
   -        47.4           - out
   -        47.5           - out
   -        47.6           - out
   -        47.7           - out
   -        47.8           - out
   -        47.9           - out
   -          48           - out
   -        48.1           - out
   -        48.2           - out
   -        48.3           - out
   -        48.4           - out
   -        48.5           - out
   -        48.6           - out
   -        48.7           - out
   -        48.8           - out
   -        48.9           - out
   -          49           - out
   -        49.1           - out
   -        49.2           - out
   -        49.3           - out
   -        49.4           - out
   -        49.5           - out
   -        49.6           - out
   -        49.7           - out
   -        49.8           - out
   -        49.9           - out
   -          50           - out
   -        50.1           - out
   -        50.2           - out
   -        50.3           - out
   -        50.4           - out
   -        50.5           - out
   -        50.6           - out
   -        50.7           - out
   -        50.8           - out
   -        50.9           - out
   -          51           - out
   -        51.1           - out
   -        51.2           - out
   -        51.3           - out
  11       51.36          12       23   14   23     1     0      0  1.2e-13  9.8e-14
   -        51.4           - out
   -        51.5           - out
   -        51.6           - out
   -        51.7           - out
   -        51.8           - out
   -        51.9           - out
   -          52           - out
   -        52.1           - out
   -        52.2           - out
   -        52.3           - out
   -        52.4           - out
   -        52.5           - out
   -        52.6           - out
   -        52.7           - out
   -        52.8           - out
   -        52.9           - out
   -          53           - out
   -        53.1           - out
   -        53.2           - out
   -        53.3           - out
   -        53.4           - out
   -        53.5           - out
   -        53.6           - out
   -        53.7           - out
   -        53.8           - out
   -        53.9           - out
   -          54           - out
   -        54.1           - out
   -        54.2           - out
   -        54.3           - out
   -        54.4           - out
   -        54.5           - out
   -        54.6           - out
   -        54.7           - out
   -        54.8           - out
   -        54.9           - out
   -          55           - out
   -        55.1           - out
   -        55.2           - out
   -        55.3           - out
   -        55.4           - out
   -        55.5           - out
   -        55.6           - out
   -        55.7           - out
   -        55.8           - out
   -        55.9           - out
   -          56           - out
   -        56.1           - out
   -        56.2           - out
   -        56.3           - out
   -        56.4           - out
   -        56.5           - out
   -        56.6           - out
   -        56.7           - out
   -        56.8           - out
   -        56.9           - out
   -          57           - out
   -        57.1           - out
   -        57.2           - out
   -        57.3           - out
   -        57.4           - out
   -        57.5           - out
   -        57.6           - out
   -        57.7           - out
   -        57.8           - out
   -        57.9           - out
   -          58           - out
   -        58.1           - out
   -        58.2           - out
   -        58.3           - out
   -        58.4           - out
   -        58.5           - out
   -        58.6           - out
   -        58.7           - out
   -        58.8           - out
   -        58.9           - out
   -          59           - out
   -        59.1           - out
   -        59.2           - out
   -        59.3           - out
   -        59.4           - out
   -        59.5           - out
   -        59.6           - out
   -        59.7           - out
   -        59.8           - out
   -        59.9           - out
   -          60           - out
   -        60.1           - out
   -        60.2           - out
   -        60.3           - out
   -        60.4           - out
   -        60.5           - out
   -        60.6           - out
   -        60.7           - out
   -        60.8           - out
   -        60.9           - out
   -          61           - out
   -        61.1           - out
   -        61.2           - out
   -        61.3           - out
   -        61.4           - out
   -        61.5           - out
   -        61.6           - out
   -        61.7           - out
   -        61.8           - out
   -        61.9           - out
   -          62           - out
   -        62.1           - out
   -        62.2           - out
   -        62.3           - out
   -        62.4           - out
   -        62.5           - out
   -        62.6           - out
   -        62.7           - out
   -        62.8           - out
   -        62.9           - out
   -          63           - out
   -        63.1           - out
   -        63.2           - out
   -        63.3           - out
  12       63.36          12       25   15   25     1     0      0  1.1e-13  8.7e-14
   -        63.4           - out
   -        63.5           - out
   -        63.6           - out
   -        63.7           - out
   -        63.8           - out
   -        63.9           - out
   -          64           - out
   -        64.1           - out
   -        64.2           - out
   -        64.3           - out
   -        64.4           - out
   -        64.5           - out
   -        64.6           - out
   -        64.7           - out
   -        64.8           - out
   -        64.9           - out
   -          65           - out
   -        65.1           - out
   -        65.2           - out
   -        65.3           - out
   -        65.4           - out
   -        65.5           - out
   -        65.6           - out
   -        65.7           - out
   -        65.8           - out
   -        65.9           - out
   -          66           - out
   -        66.1           - out
   -        66.2           - out
   -        66.3           - out
   -        66.4           - out
   -        66.5           - out
   -        66.6           - out
   -        66.7           - out
   -        66.8           - out
   -        66.9           - out
   -          67           - out
   -        67.1           - out
   -        67.2           - out
   -        67.3           - out
   -        67.4           - out
   -        67.5           - out
   -        67.6           - out
   -        67.7           - out
   -        67.8           - out
   -        67.9           - out
   -          68           - out
   -        68.1           - out
   -        68.2           - out
   -        68.3           - out
   -        68.4           - out
   -        68.5           - out
   -        68.6           - out
   -        68.7           - out
   -        68.8           - out
   -        68.9           - out
   -          69           - out
   -        69.1           - out
   -        69.2           - out
   -        69.3           - out
   -        69.4           - out
   -        69.5           - out
   -        69.6           - out
   -        69.7           - out
   -        69.8           - out
   -        69.9           - out
   -          70           - out
   -        70.1           - out
   -        70.2           - out
   -        70.3           - out
   -        70.4           - out
   -        70.5           - out
   -        70.6           - out
   -        70.7           - out
   -        70.8           - out
   -        70.9           - out
   -          71           - out
   -        71.1           - out
   -        71.2           - out
   -        71.3           - out
   -        71.4           - out
   -        71.5           - out
   -        71.6           - out
   -        71.7           - out
   -        71.8           - out
   -        71.9           - out
   -          72           - out
   -        72.1           - out
   -        72.2           - out
   -        72.3           - out
   -        72.4           - out
   -        72.5           - out
   -        72.6           - out
   -        72.7           - out
   -        72.8           - out
   -        72.9           - out
   -          73           - out
   -        73.1           - out
   -        73.2           - out
   -        73.3           - out
   -        73.4           - out
   -        73.5           - out
   -        73.6           - out
   -        73.7           - out
   -        73.8           - out
   -        73.9           - out
   -          74           - out
   -        74.1           - out
   -        74.2           - out
   -        74.3           - out
   -        74.4           - out
   -        74.5           - out
   -        74.6           - out
   -        74.7           - out
   -        74.8           - out
   -        74.9           - out
   -          75           - out
   -        75.1           - out
   -        75.2           - out
   -        75.3           - out
  13       75.36          12       26   16   26     2     0      0  3.9e-14  6.7e-14
   -        75.4           - out
   -        75.5           - out
   -        75.6           - out
   -        75.7           - out
   -        75.8           - out
   -        75.9           - out
   -          76           - out
   -        76.1           - out
   -        76.2           - out
   -        76.3           - out
   -        76.4           - out
   -        76.5           - out
   -        76.6           - out
   -        76.7           - out
   -        76.8           - out
   -        76.9           - out
   -          77           - out
   -        77.1           - out
   -        77.2           - out
   -        77.3           - out
   -        77.4           - out
   -        77.5           - out
   -        77.6           - out
   -        77.7           - out
   -        77.8           - out
   -        77.9           - out
   -          78           - out
   -        78.1           - out
   -        78.2           - out
   -        78.3           - out
   -        78.4           - out
   -        78.5           - out
   -        78.6           - out
   -        78.7           - out
   -        78.8           - out
   -        78.9           - out
   -          79           - out
   -        79.1           - out
   -        79.2           - out
   -        79.3           - out
   -        79.4           - out
   -        79.5           - out
   -        79.6           - out
   -        79.7           - out
   -        79.8           - out
   -        79.9           - out
   -          80           - out
   -        80.1           - out
   -        80.2           - out
   -        80.3           - out
   -        80.4           - out
   -        80.5           - out
   -        80.6           - out
   -        80.7           - out
   -        80.8           - out
   -        80.9           - out
   -          81           - out
   -        81.1           - out
   -        81.2           - out
   -        81.3           - out
   -        81.4           - out
   -        81.5           - out
   -        81.6           - out
   -        81.7           - out
   -        81.8           - out
   -        81.9           - out
   -          82           - out
   -        82.1           - out
   -        82.2           - out
   -        82.3           - out
   -        82.4           - out
   -        82.5           - out
   -        82.6           - out
   -        82.7           - out
   -        82.8           - out
   -        82.9           - out
   -          83           - out
   -        83.1           - out
   -        83.2           - out
   -        83.3           - out
   -        83.4           - out
   -        83.5           - out
   -        83.6           - out
   -        83.7           - out
   -        83.8           - out
   -        83.9           - out
   -          84           - out
   -        84.1           - out
   -        84.2           - out
   -        84.3           - out
   -        84.4           - out
   -        84.5           - out
   -        84.6           - out
   -        84.7           - out
   -        84.8           - out
   -        84.9           - out
   -          85           - out
   -        85.1           - out
   -        85.2           - out
   -        85.3           - out
   -        85.4           - out
   -        85.5           - out
   -        85.6           - out
   -        85.7           - out
   -        85.8           - out
   -        85.9           - out
   -          86           - out
   -        86.1           - out
   -        86.2           - out
   -        86.3           - out
   -        86.4           - out
   -        86.5           - out
   -        86.6           - out
   -        86.7           - out
   -        86.8           - out
   -        86.9           - out
   -          87           - out
   -        87.1           - out
   -        87.2           - out
   -        87.3           - out
  14       87.36          12       27   17   27     2     0      0  1.9e-14  2.5e-14
   -        87.4           - out
   -        87.5           - out
   -        87.6           - out
   -        87.7           - out
   -        87.8           - out
   -        87.9           - out
   -          88           - out
   -        88.1           - out
   -        88.2           - out
   -        88.3           - out
   -        88.4           - out
   -        88.5           - out
   -        88.6           - out
   -        88.7           - out
   -        88.8           - out
   -        88.9           - out
   -          89           - out
   -        89.1           - out
   -        89.2           - out
   -        89.3           - out
   -        89.4           - out
   -        89.5           - out
   -        89.6           - out
   -        89.7           - out
   -        89.8           - out
   -        89.9           - out
   -          90           - out
   -        90.1           - out
   -        90.2           - out
   -        90.3           - out
   -        90.4           - out
   -        90.5           - out
   -        90.6           - out
   -        90.7           - out
   -        90.8           - out
   -        90.9           - out
   -          91           - out
   -        91.1           - out
   -        91.2           - out
   -        91.3           - out
   -        91.4           - out
   -        91.5           - out
   -        91.6           - out
   -        91.7           - out
   -        91.8           - out
   -        91.9           - out
   -          92           - out
   -        92.1           - out
   -        92.2           - out
   -        92.3           - out
   -        92.4           - out
   -        92.5           - out
   -        92.6           - out
   -        92.7           - out
   -        92.8           - out
   -        92.9           - out
   -          93           - out
   -        93.1           - out
   -        93.2           - out
   -        93.3           - out
   -        93.4           - out
   -        93.5           - out
   -        93.6           - out
   -        93.7           - out
   -        93.8           - out
   -        93.9           - out
   -          94           - out
   -        94.1           - out
   -        94.2           - out
   -        94.3           - out
   -        94.4           - out
   -        94.5           - out
   -        94.6           - out
   -        94.7           - out
   -        94.8           - out
   -        94.9           - out
   -          95           - out
   -        95.1           - out
   -        95.2           - out
   -        95.3           - out
   -        95.4           - out
   -        95.5           - out
   -        95.6           - out
   -        95.7           - out
   -        95.8           - out
   -        95.9           - out
   -          96           - out
   -        96.1           - out
   -        96.2           - out
   -        96.3           - out
   -        96.4           - out
   -        96.5           - out
   -        96.6           - out
   -        96.7           - out
   -        96.8           - out
   -        96.9           - out
   -          97           - out
   -        97.1           - out
   -        97.2           - out
   -        97.3           - out
   -        97.4           - out
   -        97.5           - out
   -        97.6           - out
   -        97.7           - out
   -        97.8           - out
   -        97.9           - out
   -          98           - out
   -        98.1           - out
   -        98.2           - out
   -        98.3           - out
   -        98.4           - out
   -        98.5           - out
   -        98.6           - out
   -        98.7           - out
   -        98.8           - out
   -        98.9           - out
   -          99           - out
   -        99.1           - out
   -        99.2           - out
   -        99.3           - out
  15       99.36          12       28   18   28     2     0      0  3.2e-14  3.5e-14
   -        99.4           - out
   -        99.5           - out
   -        99.6           - out
   -        99.7           - out
   -        99.8           - out
   -        99.9           - out
   -         100           - out
   -       100.1           - out
   -       100.2           - out
   -       100.3           - out
   -       100.4           - out
   -       100.5           - out
   -       100.6           - out
   -       100.7           - out
   -       100.8           - out
   -       100.9           - out
   -         101           - out
   -       101.1           - out
   -       101.2           - out
   -       101.3           - out
   -       101.4           - out
   -       101.5           - out
   -       101.6           - out
   -       101.7           - out
   -       101.8           - out
   -       101.9           - out
   -         102           - out
   -       102.1           - out
   -       102.2           - out
   -       102.3           - out
   -       102.4           - out
   -       102.5           - out
   -       102.6           - out
   -       102.7           - out
   -       102.8           - out
   -       102.9           - out
   -         103           - out
   -       103.1           - out
   -       103.2           - out
   -       103.3           - out
   -       103.4           - out
   -       103.5           - out
   -       103.6           - out
   -       103.7           - out
   -       103.8           - out
   -       103.9           - out
   -         104           - out
   -       104.1           - out
   -       104.2           - out
   -       104.3           - out
   -       104.4           - out
   -       104.5           - out
   -       104.6           - out
   -       104.7           - out
   -       104.8           - out
   -       104.9           - out
   -         105           - out
   -       105.1           - out
   -       105.2           - out
   -       105.3           - out
   -       105.4           - out
   -       105.5           - out
   -       105.6           - out
   -       105.7           - out
   -       105.8           - out
   -       105.9           - out
   -         106           - out
   -       106.1           - out
   -       106.2           - out
   -       106.3           - out
   -       106.4           - out
   -       106.5           - out
   -       106.6           - out
   -       106.7           - out
   -       106.8           - out
   -       106.9           - out
   -         107           - out
   -       107.1           - out
   -       107.2           - out
   -       107.3           - out
   -       107.4           - out
   -       107.5           - out
   -       107.6           - out
   -       107.7           - out
   -       107.8           - out
   -       107.9           - out
   -         108           - out
   -       108.1           - out
   -       108.2           - out
   -       108.3           - out
   -       108.4           - out
   -       108.5           - out
   -       108.6           - out
   -       108.7           - out
   -       108.8           - out
   -       108.9           - out
   -         109           - out
   -       109.1           - out
   -       109.2           - out
   -       109.3           - out
   -       109.4           - out
   -       109.5           - out
   -       109.6           - out
   -       109.7           - out
   -       109.8           - out
   -       109.9           - out
   -         110           - out
   -       110.1           - out
   -       110.2           - out
   -       110.3           - out
   -       110.4           - out
   -       110.5           - out
   -       110.6           - out
   -       110.7           - out
   -       110.8           - out
   -       110.9           - out
   -         111           - out
   -       111.1           - out
   -       111.2           - out
   -       111.3           - out
  16      111.36          12       29   19   29     2     0      0  4.4e-14  4.5e-14
   -       111.4           - out
   -       111.5           - out
   -       111.6           - out
   -       111.7           - out
   -       111.8           - out
   -       111.9           - out
   -         112           - out
   -       112.1           - out
   -       112.2           - out
   -       112.3           - out
   -       112.4           - out
   -       112.5           - out
   -       112.6           - out
   -       112.7           - out
   -       112.8           - out
   -       112.9           - out
   -         113           - out
   -       113.1           - out
   -       113.2           - out
   -       113.3           - out
   -       113.4           - out
   -       113.5           - out
   -       113.6           - out
   -       113.7           - out
   -       113.8           - out
   -       113.9           - out
   -         114           - out
   -       114.1           - out
   -       114.2           - out
   -       114.3           - out
   -       114.4           - out
   -       114.5           - out
   -       114.6           - out
   -       114.7           - out
   -       114.8           - out
   -       114.9           - out
   -         115           - out
   -       115.1           - out
   -       115.2           - out
   -       115.3           - out
   -       115.4           - out
   -       115.5           - out
   -       115.6           - out
   -       115.7           - out
   -       115.8           - out
   -       115.9           - out
   -         116           - out
   -       116.1           - out
   -       116.2           - out
   -       116.3           - out
   -       116.4           - out
   -       116.5           - out
   -       116.6           - out
   -       116.7           - out
   -       116.8           - out
   -       116.9           - out
   -         117           - out
   -       117.1           - out
   -       117.2           - out
   -       117.3           - out
   -       117.4           - out
   -       117.5           - out
   -       117.6           - out
   -       117.7           - out
   -       117.8           - out
   -       117.9           - out
   -         118           - out
   -       118.1           - out
   -       118.2           - out
   -       118.3           - out
   -       118.4           - out
   -       118.5           - out
   -       118.6           - out
   -       118.7           - out
   -       118.8           - out
   -       118.9           - out
   -         119           - out
   -       119.1           - out
   -       119.2           - out
   -       119.3           - out
   -       119.4           - out
   -       119.5           - out
   -       119.6           - out
   -       119.7           - out
   -       119.8           - out
   -       119.9           - out
   -         120           - out
  17      123.36          12       30   20   30     2     0      0  1.3e-14  4.5e-14
Time stepping completed
```

##### fc1

General

| **Description** | **Value** |
| --- | --- |
| Linear solver |  |

Method and termination

| **Description** | **Value** |
| --- | --- |
| Damping factor | 0.9 |
| Jacobian update | Once per time step |
| Stabilization and acceleration | Anderson acceleration |
| Dimension of iteration space | 5 |
| Mixing parameter | 0.9 |
| Iteration delay | 1 |

##### ht

General

| **Description** | **Value** |
| --- | --- |
| Solver | PARDISO |
| Pivoting perturbation | 1E-13 |

# 4 Result

## 4.1 Dataset

### 4.1.1 Study

Solution

| **Description** | **Value** |
| --- | --- |
| Solution |  |
| Component | comp1 |

### 4.1.2 Probe Solution

Solution

| **Description** | **Value** |
| --- | --- |
| Solution |  |
| Component | comp1 |

### 4.1.3 Boundary Probe 2

Selection

| Geometric entity level | Boundary |
| --- | --- |
| Selection | Geometry geom1: Dimension 2: Boundaries 108–109, 121, 146, 156 |

Data

| **Description** | **Value** |
| --- | --- |
| Dataset |  |

Settings

| **Description** | **Value** |
| --- | --- |
| Method | Integration |
| Integration order | 4 |
| Integration order | On |

## 4.2 Derived Values

Data

| **Description** | **Value** |
| --- | --- |
| Dataset |  |

Expressions

| **Expression** | **Unit** |
| --- | --- |
| T | °C |

## 4.3 table

### 4.3.1 Evaluation 3D

| **x** | **y** | **z** | **Value** |
| --- | --- | --- | --- |
| -0.093808 | 0.34626 | -0.001522 | 20.006 |
| -0.0913 | 0.34504 | -0.0042867 | 20.021 |
| -0.080931 | 0.31661 | -0.018556 | 26.207 |
| -0.081313 | 0.31784 | -0.020544 | 25.706 |
| -0.073342 | 0.33806 | -0.017707 | 25.918 |
| -0.070591 | 0.33849 | -0.011799 | 26.216 |
| -0.078688 | 0.31022 | -0.022788 | 26.329 |
| -0.091615 | 0.32198 | -0.0032006 | 20.422 |
| -0.093896 | 0.32512 | -0.0080378 | 20.447 |
| -0.089484 | 0.31947 | -0.015455 | 22.395 |
| -0.091748 | 0.32148 | -0.013901 | 21.326 |
| -0.09307 | 0.32329 | -0.011788 | 20.83 |
| -0.09334 | 0.32377 | -0.011309 | 20.736 |
| -0.092768 | 0.32275 | -0.01215 | 20.928 |
| -0.0908 | 0.32046 | -0.011898 | 21.553 |
| -0.089509 | 0.31934 | -0.013561 | 22.304 |
| -0.093401 | 0.32406 | -0.0086887 | 20.574 |
| -0.094615 | 0.32897 | -0.0038891 | 20.181 |
| -0.095027 | 0.33018 | -0.015429 | 20.366 |
| -0.080339 | 0.3428 | -0.0053862 | 21.03 |
| -0.090154 | 0.34624 | 4.3419E-4 | 20.021 |
| -0.075485 | 0.33999 | -0.012463 | 28.06 |
| -0.094679 | 0.3269 | -0.0111 | 20.429 |
| -0.091056 | 0.32148 | -0.019374 | 21.484 |
| -0.093982 | 0.32997 | -0.022408 | 20.373 |
| -0.092127 | 0.32233 | -0.0059396 | 20.592 |
| -0.064649 | 0.31342 | 0.0019635 | 20.052 |
| -0.069909 | 0.30981 | -0.027509 | 36.671 |
| -0.070293 | 0.30965 | -0.026283 | 36.642 |
| -0.068412 | 0.31049 | -0.02862 | 36.712 |
| -0.06629 | 0.31163 | -0.028497 | 36.714 |
| -0.06677 | 0.31088 | -0.026978 | 36.746 |
| -0.069527 | 0.30984 | -0.027324 | 36.687 |
| -0.068731 | 0.30999 | -0.027074 | 36.716 |
| -0.068731 | 0.30999 | -0.027074 | 36.716 |
| -0.068731 | 0.30999 | -0.027074 | 36.716 |
| -0.06758 | 0.31044 | -0.02709 | 36.741 |
| -0.083306 | 0.32367 | 0.023397 | 20 |
| -0.081799 | 0.31944 | 0.037346 | 20 |
| -0.091426 | 0.32221 | -9.6279E-4 | 20.275 |

## 4.4 Plot Groups

### 4.4.1 ht

Volume: Temperature (°C) Isosurface: Temperature (°C)
